# Deep learning-based encryption scheme for medical images using DCGAN and virtual planet domain

**DOI:** 10.1038/s41598-024-84186-6

**Published:** 2025-01-07

**Authors:** Manish Kumar, Aneesh Sreevallabh Chivukula, Gunjan Barua

**Affiliations:** 1 Department of Mathematics, Birla Institute of Technology and Science-Pilani, Hyderabad Campus, Hyderabad, 500078 Telangana India; 2 Department of Computer Science and Information Systems, Birla Institute of Technology and Science-Pilani, Hyderabad Campus, Hyderabad, 500078 India

**Keywords:** DCGAN, Chaotic map, VPD, Encryption, Decryption, Computer science, Applied mathematics, Information technology, Machine learning, Image processing

## Abstract

The motivation for this article stems from the fact that medical image security is crucial for maintaining patient confidentiality and protecting against unauthorized access or manipulation. This paper presents a novel encryption technique that integrates the Deep Convolutional Generative Adversarial Networks (DCGAN) and Virtual Planet Domain (VPD) approach to enhance the protection of medical images. The method uses a Deep Learning (DL) framework to generate a decoy image, which forms the basis for generating encryption keys using a timestamp, nonce, and 1-D Exponential Chebyshev map (1-DEC). Experimental results validate the efficacy of the approach in safeguarding medical images from various security threats, including unauthorized access, tampering, and adversarial attacks. The randomness of the keys and encrypted images are demonstrated through the National Institute of Standards and Technology (NIST) SP 800-22 Statistical test suite provided in Tables 4 and 14, respectively. The robustness against key sensitivity, noise, cropping attacks, and adversarial attacks are shown in Figs. 15–18, 22–23, and 24. The data presented in Tables 5, 6, and 7 shows the proposed algorithm is robust and efficient in terms of time and key space complexity. Security analysis results are shown (such as histogram plots in Figs. 11–14 and correlation plots in Figs. 19–21). Information Entropy ($7.9993 \pm 0.0001$), correlation coefficient ($\pm 0.09$), Mean Square Error (MSE) ($4166.3107 \pm 1645.2980$), Peak Signal to Noise Ratio (PSNR) ($12.2643 \pm 1.7032$), Number of Pixel Change Rate (NPCR) ($99.60\% \pm 0.2\%$), and Unified Average Changing Intensity (UACI) ($33.47\% \pm 0.1\%$) underscore the high security and reliability of the encrypted images, are shown in Tables 8–11. Further, statistical NPCR and UACI are calculated in Tables 12 and 13, respectively. The proposed algorithm is also compared with existing algorithms, and compared values are provided in Table 15. The data presented in Tables 3–15 suggest that the proposed algorithm can opt for practical use.

## Introduction

Medical image encryption is vital for maintaining the confidentiality and integrity of sensitive medical data, ensuring that it remains accessible only to authorized individuals. Protecting the privacy and integrity of medical images is becoming increasingly important as medical imaging technology advances, improving diagnostic accuracy and guiding treatment plans. Medical images, such as X-rays, Magnetic Resonance Images (MRI), and Computed Tomography (CT) scans, contain highly personal information that could be misused if accessed by unauthorized parties, leading to privacy violations and even potential financial or reputational harm to patients. Furthermore, the precision of medical images is essential for correct diagnosis and treatment, as unauthorized alteration or corruption of these images could lead to incorrect treatment plans, potentially endangering patient health.

The growing adoption of telemedicine and cloud-based healthcare systems has further emphasized the need for secure data transmission and storage. Encryption ensures that medical images transmitted over networks or stored in cloud platforms remain protected from breaches and cyber threats while facilitating research and collaboration by safeguarding patient privacy.

In the literature, various sophisticated encryption algorithms have been developed in^1–24^ and references therein to secure medical images, each with strengths and limitations. DNA encoding offers robust encryption by leveraging genetic sequence patterns but is constrained by the need for specialized setups, limiting its practicality^1–4^. Chaotic maps, often combined with substitution boxes, are valued for their sensitivity to initial conditions and nonlinearity, which are core features of chaos theory, making it ideal for cryptographic applications^5–7^. Digital chaos, modeled as iterative dynamical systems, can be implemented across one-dimensional (1D), two-dimensional (2D), and hyperdimensional (HD) frameworks. These have been applied to domains such as secure communication^25^, pseudo-random number generation^26^, image encryption^27,28^, and video encryption^29,30^. While higher-dimensional chaotic systems offer larger key spaces and more complex behaviors for enhanced security, they also incur higher computational costs and increased encryption time. Conversely, 1D chaotic systems are computationally efficient but may exhibit limitations like dynamic degeneracy and non-uniform distributions, potentially reducing their unpredictability. Techniques such as zigzag XORing^8–11^ and block-based methods like AES^12,13^ provide reliable security, but AES struggles with the strong pixel correlations in medical images, leading to inefficiencies. Deep learning (DL) has revolutionized encryption, enabling models like Convolutional Neural Networks (CNNs) to dynamically learn encryption patterns, manage keys, and detect anomalies, enhancing adaptability and resilience against attacks^31–35^. Methods such as DeepEDN^36^, EncipherGAN^15^, and ResNet-based approaches ^16 ^integrate DL with traditional cryptographic techniques for enhanced robustness. Dynamic key generation methods that leverage neural networks have also been proposed, which adapt to the needs of the dynamic and scalable encryption necessary for modern medical imaging systems in^19^. Novel DL applications, including direct image encryption using DCT coefficients^37^, DCGANs with quaternion mathematics^17^, and GANs generating synthetic images for privacy^18^, highlight the field’s innovation. However, these methods often face challenges like high computational demands and scalability issues, particularly during encryption and decryption. Hybrid approaches combining chaotic maps, GANs, and Huffman compression^20^, as well as 4D chaotic systems for critical area protection^21^, offer improved multilayered security but remain computationally expensive. Addressing these limitations requires balancing computational efficiency, adaptability, and security, paving the way for more practical and scalable encryption solutions in medical imaging applications.

Motivated by the limitations and challenges described above, we propose a novel encryption method for medical images that employs a DCGAN associated with VPD to increase the efficiency of cryptographic operations by segregating the key generation phase from the encryption and decryption routines, thus reducing computational load. Furthermore, chaotic maps are utilized to derive robust encryption keys, with security enhancements achieved through techniques such as VPD, 3D pixel intershuffling, and zigzag XORing. The proposed methodology seeks to balance enhanced security with computational efficiency, addressing contemporary cryptographic application evolving needs and significantly complicating unauthorized data access or manipulation.

## Organization of the work

"Preliminaries" provides a detailed discussion of the DCGAN, 1-DEC, and VPD. In "Key generation procedure", the key generation procedure is explained along with pseudocode. "Proposed encryption and decryption process" presents the proposed encryption and decryption algorithms and pseudocodes, including encryption and decryption results. In "Security analysis", security analysis reports of the proposed algorithm are given. We provide a comparative analysis with existing methods in " Comparative analysis ". The last section concludes the proposed work.

## Preliminaries

In this section, we discuss three main ingredients (DCGAN, 1-DEC, and VPD) used to generate random keys and employ them during the encryption process to provide robust support for the proposed novel encryption algorithm.

### DCGAN

The GAN^38^ is a deep learning architecture where two neural networks, the generator $G$ and the discriminator $D$, are involved in a game theoretic interaction to produce realistic synthetic data. The generator receives a noise vector $z$ as input, typically sampled from a prior distribution such as either the uniform or the Gaussian noise distribution. The generator’s objective is to transform $z$ into synthetic data that closely matches the training data. The discriminator takes samples drawn from the given training data $x$ and from the generated synthetic data $G(z)$ as input and attempts to classify them as real (label 1) or fake (label 0). In a min-max game between the two models with opposing objectives - the discriminator aims to get better at distinguishing between real and fake images, whereas the generator learns to produce fake images that look like the original data, ultimately ‘fooling’ the discriminator into classifying a fake image as real.

As a novel image generation model, GANs have gained popularity among researchers, leading to the development of numerous generative deep learning frameworks. Key breakthroughs include CGAN^39^for stable training with category labels, DCGAN with improved training approaches^40^, and subsequent advancements such as Pix2Pix^41^, CycleGAN^42^, StyleGAN^43^, and BigGAN^44^, which have optimized the image generation in a variety of real-world settings. DCGANs^45^ integrated CNNs into the GAN architecture. Applications in image generation^46^ reveal that DCGAN has produced images that are up to twice as high in quality compared to those generated by standard GANs, along with significantly greater diversity in images, establishing themselves as powerful techniques for generative learning. Its architecture is versatile enough to generate specific types of decoy images, enabling the model to be tailored to meet the unique needs of an encryption scheme.

In^47^, various deep learning techniques used in image encryption are surveyed. The use of deep learning enhances the encryption system’s security, particularly against plaintext attacks, through non-linearity in cryptanalysis. One common approach is style transfer, where GANs and CycleGANs are used to convert plaintext images into ciphertexts. The encryption network is trained to generate ciphertext images, while the decryption network aims to restore the original image. Here, the parameters of the networks act as the secret keys, resulting in a large key space that increases security. In a medical imaging application for diagnosis and treatments based on brain MRI and CT scan data^36^, introduces “DLEDNet,” a deep learning architecture where encryption and decryption networks, along with a discriminator neural network, combine to enhance the encryption performance. Similarly, in^15^, a cycle-GAN-based algorithm is developed for image encryption, and tested on medical datasets. Compared to chaotic-map-based encryption methods, which are vulnerable to phase-space-reconstruction attacks, the proposed model’s nonlinearity enhances the encryption system’s security. Style transfer approaches with additional diffusion properties, as seen in^48^, improve the robustness of encryption systems. Here, a cover image is used to disguise the plaintext, followed by a domain transformation using CycleGAN, combining steganography with deep learning. Chaotic sequences also play a significant role in deep learning-based encryption, where convolutional kernels are generated by chaotic sequences. As noted in^37^, chaotic maps used in the diffusion of the plaintext image result in dynamic key generation, which can be combined with traditional encryption techniques like XOR^49^.

The main improvements in DCGAN over the classic GAN include replacing pooling functions with strided and fractional-strided convolutions^50^, implementing batch normalization^51 ^across all layers, removing fully connected layers to simplify the architecture, and using the Rectified Linear Unit (ReLU)^52^ and tanh activations in the generator and leaky ReLU in the discriminator, all of which contribute to improved stability and image quality. The generator takes input from a latent space (*z*) and passes it through a series of fractional-strided convolutional-transpose layers to produce synthetic images. The discriminator is a binary CNN classifier that uses strided convolutions to downsample the input images into categories (real/fake). The detailed architecture and training process are discussed in the following sections.

#### Strided convolutions and fractional-strided convolutions

Convolutional layers apply a kernel $K$, which is like a filter that slides over an input layer $X$ to extract spatial features, $Y$, called feature maps. The mathematical operation is defined as follows:

$$\begin{aligned} Y(i, j) = (X*K)(i,j) = \sum _m \sum _n X(i+m, j+n) \cdot K(m, n), \end{aligned}$$

where $i, j$ are spatial indices, and $m, n$ denote the kernel dimensions. Strided convolutions introduce a step variable (or stride) that controls the number of pixels the kernel skips while moving along the width and height of the input; downsampling the feature map can be seen in Fig. 3. This reduction in spatial dimensions helps the discriminator focus on global features, such as object shapes. A padding of zeros is added to preserve information on the border pixels. The size of the output image obtained after convolution is given by:

$$\begin{aligned}output\ size = \frac{input\ size + 2\cdot padding\ size-kernel\ size}{stride}+1.\end{aligned}$$

Fractionally-strided convolutions (also known as transposed convolutions) reverse the downsampling process to upsample input data. By inserting zeros between entries of the input feature maps, they increase the spatial dimensions of the feature map, effectively creating higher-resolution images. This upsampling is crucial for the generator to create large, high-quality images from a small latent vector. The output size is formulated as follows:

$$\begin{aligned}output\ size = s \cdot (input\ size+2\cdot padding\ size-1)+kernel\ size.\end{aligned}$$

This process can be visualized in Fig. 3.

#### Batch normalization

Batch normalization is a neural network layer, added after a hidden layer, that normalizes the output of each layer $x$ using batch mean $\mu _B$ and variance $\sigma _B^2$, along with learnable parameters $\gamma$ and $\beta$, before passing the result to the next layer (Fig. 3). This technique stabilizes training by reducing internal covariate shifts and helps the model converge faster by ensuring the inputs to each layer maintain a consistent distribution. The absence of batch normalization can cause shifts in the distribution of layer inputs during training, potentially slowing down learning and causing instability. The normalized output is given by:

$$\begin{aligned} \hat{x} = \frac{x - \mu _B}{\sqrt{\sigma _B^2+ \epsilon }}, \quad y = \gamma \hat{x} + \beta . \end{aligned}$$

#### Activation functions

Activation functions add non-linearity to neural networks, enabling them to model complex relationships between inputs and outputs. The generator uses ReLU in its hidden layers to prevent gradient saturation, while Leaky ReLU in the discriminator ensures better gradient flow for negative inputs, aiding effective learning. The tanh function scales the generator’s output to $(-1, 1)$, aligning with normalized training data, and the sigmoid function $\sigma (x)$ in the discriminator’s final layer maps outputs to probabilities $(0, 1)$, facilitating classification of real versus fake inputs. These activation functions, summarized in Table 1, play a critical role in the DCGAN architecture.

**Table 1:** Activation functions used in DCGAN neural network layers.

**Activation function**	**Formula**
Sigmoid	$\sigma (x) = \frac{1}{1 + e^{-x}}$
Hyperbolic Tangent (Tanh)	$\tanh (x) = \frac{e^x - e^{-x}}{e^x + e^{-x}}$
ReLU (Rectified Linear Unit)	$\text {ReLU}(x) = \max (0, x)$
Leaky ReLU	$$\text {Leaky ReLU}(x) = {\left\{ \begin{array}{ll} x, & x> 0 \\ ax, & x \le 0 \end{array}\right. }$$

#### Training process

The generator and discriminator are trained adversarially using the following loss functions:

- **Generator Loss:** Encourages $G$ to generate realistic images:
  $$\begin{aligned} \mathscr {L}_G = -\mathbb {E}_{z \sim p_z(z)}[\log D(G(z))]. \end{aligned}$$
- **Discriminator Loss:** Encourages $D$ to distinguish between real and fake images:
  $$\begin{aligned} \mathscr {L}_D = -\mathbb {E}_{x \sim p_{\text {data}}(x)}[\log D(x)] - \mathbb {E}_{z \sim p_z(z)}[\log (1 - D(G(z)))]. \end{aligned}$$

These functions can be combined into a unified optimization function $V(D, G)$:

$$\begin{aligned} \min _G \max _D V(D, G) = \min _G \max _D \left[ \mathbb {E}_{x \sim p_{\text {data}}(x)}[\log D(x)] + \mathbb {E}_{z \sim p_{\text {z}}(z)}[\log (1 - D(G(z)))] \right] . \end{aligned}$$

The DCGAN training alternates between optimizing $D$ for several steps and then updating $G$ for one step. In each iteration, $D$ is trained to improve its accuracy by adjusting its weights via backpropagation. After $D$ is trained and optimized for a given $G$, $G$ receives the feedback from $D$’s loss function and updates its parameters to produce data that better approximates the real data distribution. This iterative approach continues with $D$ being optimized again while $G$ is fixed. As the training progresses, $G$ indirectly learns the data distribution, gradually degrading $D$’s performance. For a fixed $G$, the optimal $D$ converges to:

$$\begin{aligned}D_G^*(x) = \frac{p_{\text {data}}(x)}{p_{\text {data}}(x) + p_g(x)},\end{aligned}$$

where $p_{\text {data}}(x)$ is the training data distribution and $p_g(x)$ is the generative distribution of samples $G(z)$. As training advances with sufficient capacity and iterations, the system converges to $D(G(z))$ approaching 0.5 for all $z$, meaning the discriminator can no longer differentiate between the two classes. This is the Nash equilibrium^53^ that occurs when $p_{\text {data}} = p_g$, indicating that the generator is successful in learning the probability distribution of the training dataset and can now be used to generate new images. In the proposed key generation, the trained generator in a DCGAN is used to generate the synthetic images that serve as the basis for creating encryption keys for input medical data. It is trained in advance, ensuring the generator is readily available for immediate key generation, thereby eliminating any delays in the key generation workflow. The unpredictable nature of synthetic images produced from noise and the complex non-linear properties of the deep CNN provides an additional level of security to the key space generated and the proposed encryption algorithm.

### Experiments

This section provides detailed descriptions of the DCGAN’s layers and parameters. Details about the DCGAN’s design, including the number of convolutional layers used, activation functions, and other important hyperparameters, like the size of the latent vector, batch size, learning rate, and optimizer settings, along with their impact on model performance, are given. This information is necessary for experimental evaluation.

#### DCGAN architecture and model parameters

**Fig. 1:**
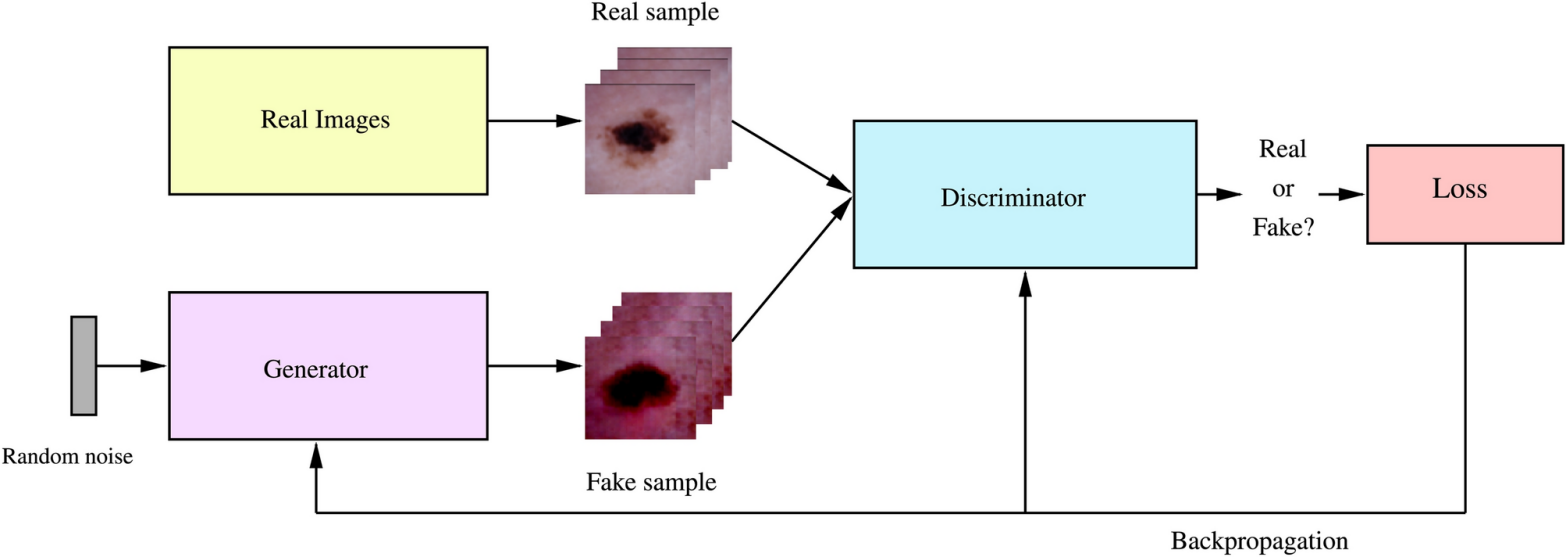
Architecture of the DCGAN consisting of generator network, discriminator network, and game theoretical min-max loss function.

**Fig. 2:**
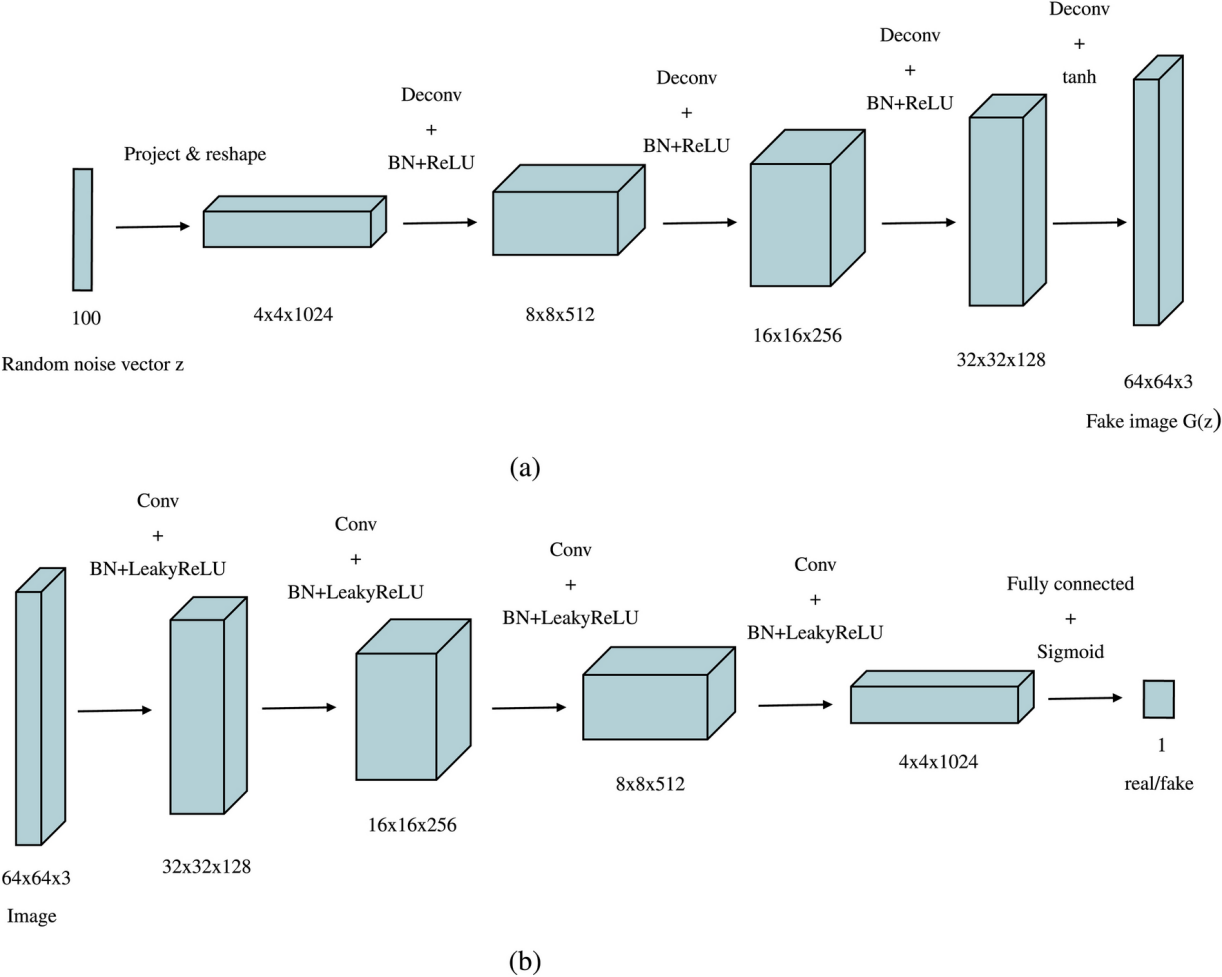
Learned representations of inputs in (**a**) the generator and (**b**) the discriminator of the DCGAN.

**Fig. 3:**
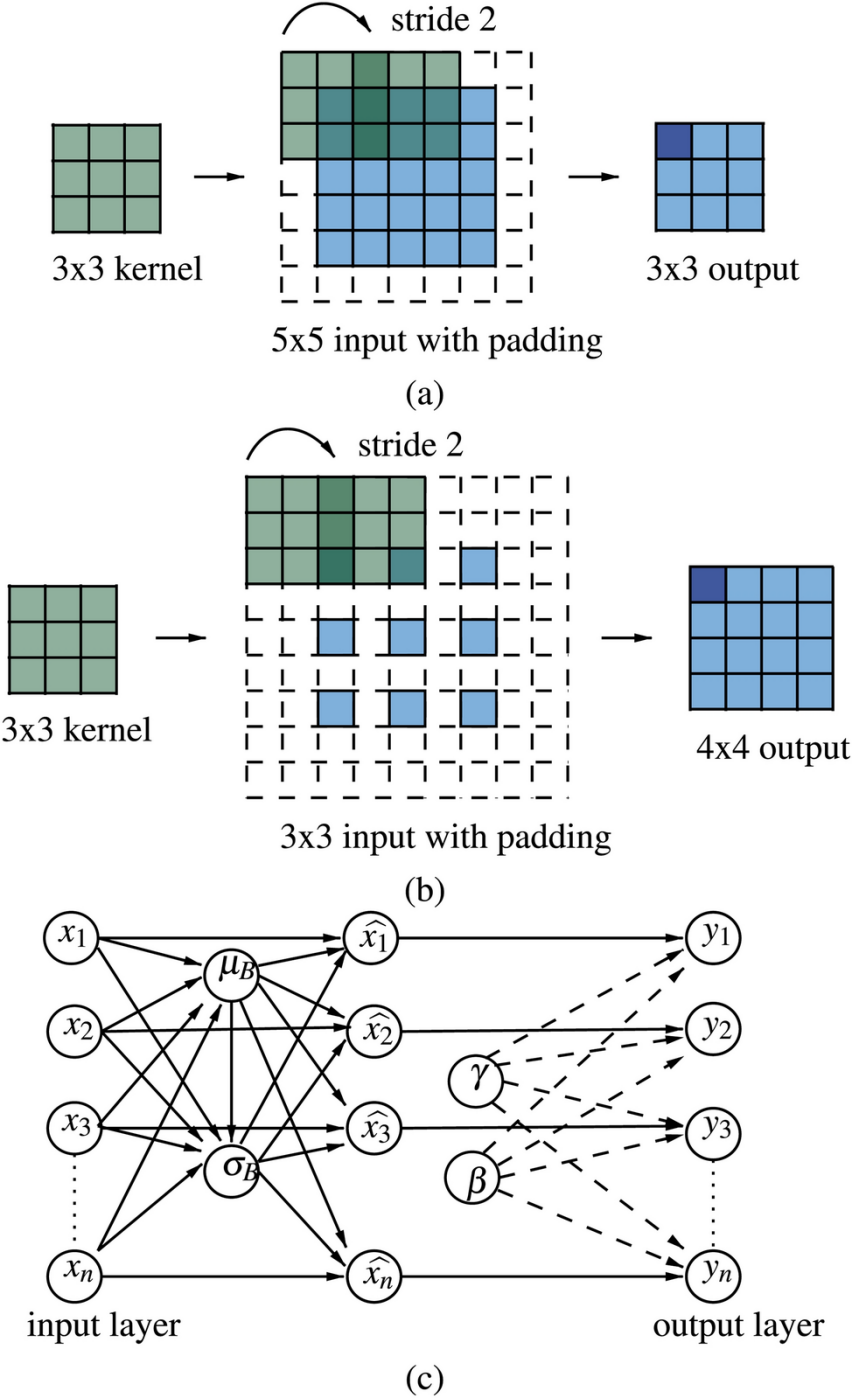
Elements of the convolutional layers: (**a**) Strided convolution operation (**b**) Fractional-strided convolution operation (**c**) Batch normalization layer.

**Fig. 4:**
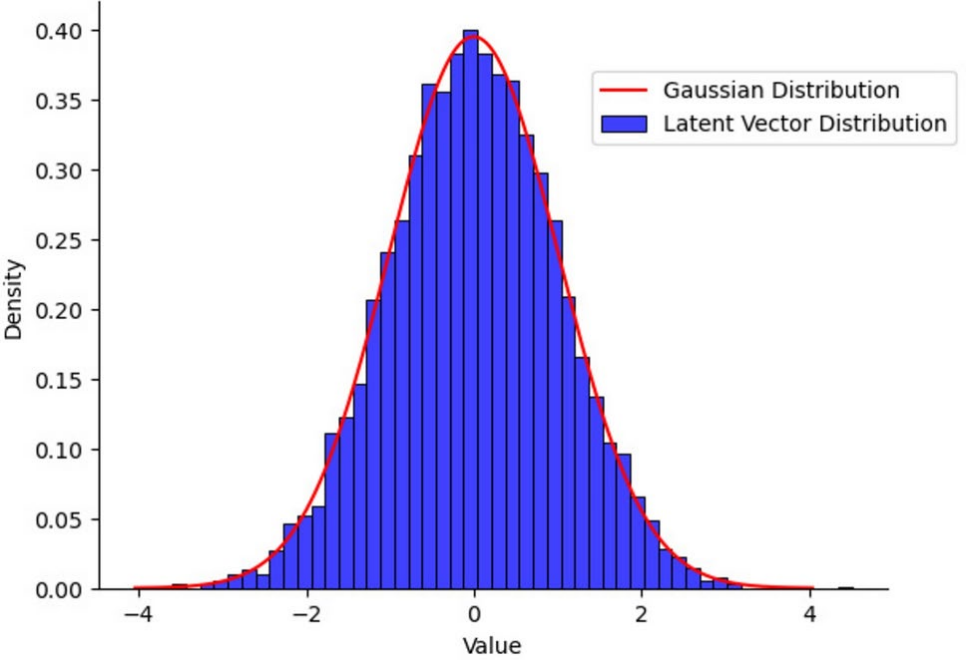
Distribution of noise vector $z$ following a Gaussian function. $z$ is the input to the generator network of the DCGAN.

The DCGAN used in our paper is designed to produce RGB images of $64 \times 64$ dimensions, consistent with the image size of the dataset utilized in this work (described in a later section). The generator begins with an input noise vector $z$ of size $1 \times 100$ sampled from a Gaussian distribution (Fig. 4). The generator’s core is organized into four groups of layers, each comprising a transposed convolution followed by batch normalization and a ReLU activation function. The final layer of the generator applies a tanh activation function to produce the output image. The input noise vector $z$ is initially reshaped into a $4 \times 4$ grid with 1024 feature maps. This vector is upsampled through the convolutional layers, gradually producing a high-resolution image with dimensions $64 \times 64 \times 3$. The deconvolution step is set to 2. Therefore, each output of a group augments fourfold the input, resulting in the output sizes of layers being $4 \times 4$, $8 \times 8$, $16 \times 16$, $32 \times 32$, and finally $64 \times 64$ in the final layer. The number of feature maps is also set to 512, 256, 128, and 64, respectively, with the final layer having 3 channels. The discriminator mirrors the generator but with strided convolutions instead of transposed convolutions. The input, either a real image or a synthetic image from the generator, is progressively downsampled through these convolutions that decrease the spatial dimensions while increasing the depth of the feature maps. The discriminator consists of three groups of layers, each containing a strided convolution followed by batch normalization (applied to all layers except the input) and a Leaky ReLU activation function. The moving step of the convolution kernel is set to 2. For an input image of dimensions $64 \times 64 \times 3$, the output sizes of the convolution layers are $32 \times 32$, $16 \times 16$, $8 \times 8$, and $4 \times 4$, with 64, 128, 256, and 512 feature maps, respectively. The final layer is a fully connected layer used for flattening feature maps into a single output neuron with a sigmoidal activation function, thus producing a probability score between 0 and 1. The score refers to how confident the model is in its classification of the input image as real or fake. Figure 1 represents the complete DCGAN architecture and training flow, Fig. 2 illustrates the learned representations of inputs in the generator and discriminator, and the layers of the neural network are shown in Fig. 3.

#### Model hyperparameter settings

In our experiments, we generally followed the default hyperparameter settings proposed in the original DCGAN publication^45^. The learning rate $\alpha$ was set to 0.0002 and the batch to 64. The Adam optimizer was selected for its adaptive learning rate. $\beta _1$, known as the First Moment Decay Rate, was set to 0.5; this helps prevent oscillations and inconsistent updates during training. The $\beta _2$ is the Second Moment Decay Rate, which controls the decay rate of the moving average for the squares of the gradients. It was kept at 0.999, which prevents the model from being too reactive to small changes in gradients and helps in more stable convergence. For the discriminator, the binary cross-entropy function was used to calculate loss. The generator network parameters are updated after one step of discriminator optimization. We have taken 100 epochs for simulation purposes. Users can select it as they see fit and use it as a secret key. The final image outputs from the generator were then used to create the initial keys for encryption and decryption in the proposed security framework.

**Fig. 5:**
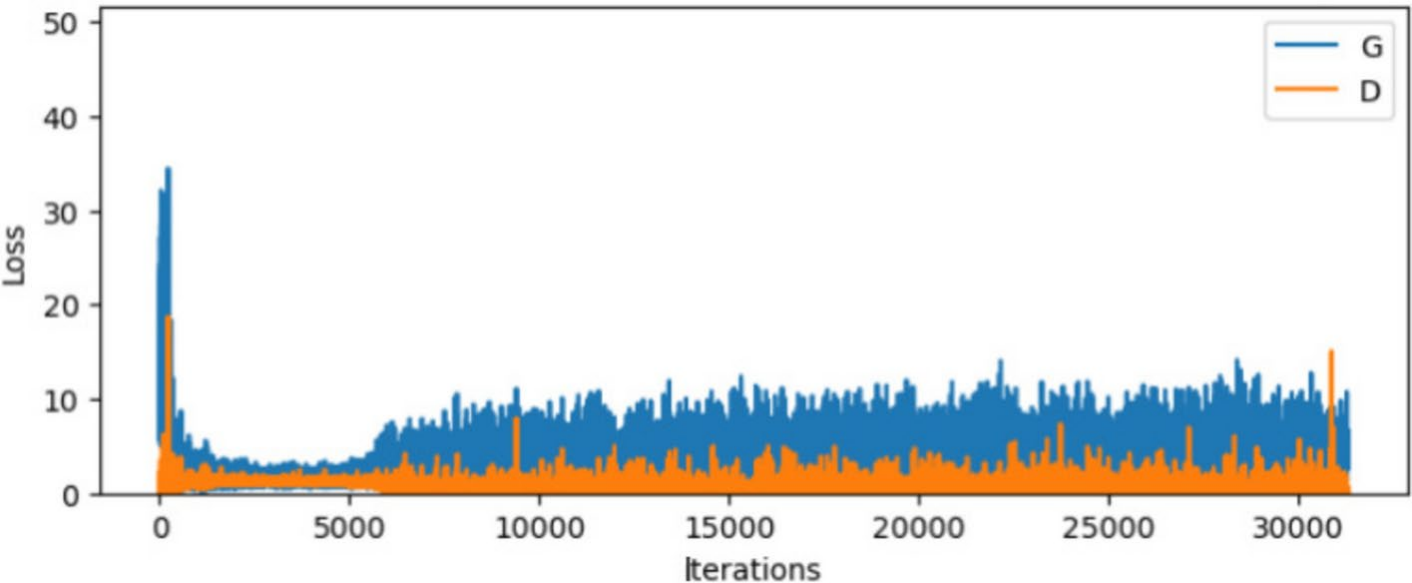
Training losses of Generator $G$ and Discriminator $D$ showing the convergence conditions of DCGAN.

**Fig. 6:**
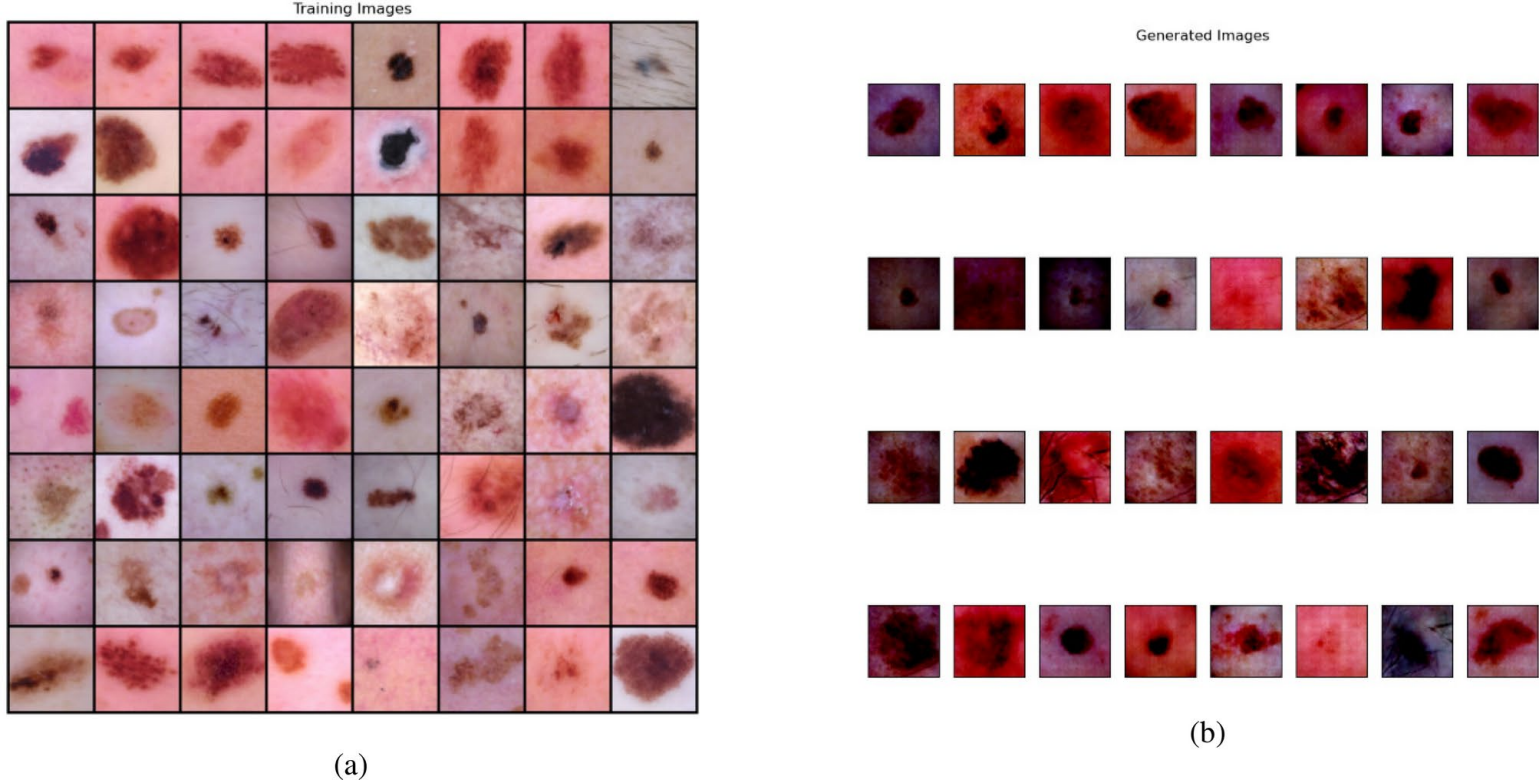
(**a**) Skin cancer images in the training dataset, (**b**) Images produced by the Generator of the DCGAN.

The generator and discriminator training loss curves across 30,000 iterations are shown in Fig. 5. During the first 3000 iterations, the discriminator’s loss rapidly decreases while the generator’s loss is initially high. Both losses stabilize after approximately 3000 iterations, indicating that the generator begins creating more realistic images, making it difficult for the discriminator to differentiate between real and fake data. After this point, the losses fluctuate but remain relatively stable, suggesting a balanced training process. This indicates the model is generating useful outputs, and further hyperparameter optimization could enhance this stability. Training could be stopped when significant divergence is observed again, around 5000 iterations, as further training might lead to instability. Figure 6 shows the comparison between training images and generated images.

#### System specifications for model training

The model training was conducted on an HPC server with an NVIDIA RTX A6000 GPU, offering 49140 MiB of dedicated memory for high-performance computational tasks. The server’s CPU is an AMD Ryzen 9 7950X 16-core Processor, supported by 125 GB of RAM and an additional 279 GB of swap memory available to handle overflow. The storage capacity includes three high-speed NVMe drives: nvme0n1p3 with 458 GB (27 GB used) and two drives (nvme2n1p1 and nvme1n1p1), each offering 3.6 TB of storage. The training environment utilized PyTorch version 2.4.0+cu121, which supports CUDA 12.1. This setup provided the computational power necessary to handle the extensive operations involved in deep learning tasks, particularly the iterative training process of GANs.

### 1-DEC

The 1-DEC was proposed in^54^ to overcome the traditional 1-Dimensional Chebyshev Chaotic Map (1-DCCM), tent map, and logistic map that encounter limitations related to its control parameters, empty intervals, and non-uniform state distribution. Adding an exponential function to the 1-DCCM greatly improved its performance, and it is called 1-DEC. The 1-DEC map is expressed in Eq. (1) as follows:

1 $$\begin{aligned} y_{n+1} = 1 - 2 \left( \cos \left( \arccos (y_n) \cdot e^{|\mu |}\arccos (y_n)\right) \right) ^2, \end{aligned}$$

where $\mu \in \mathbb {R}$ is the control parameter of the 1-DEC, and $y_{n+1} (-1 \le y_{n+1} \le 1)$represents the output sequence generated by the map. The sequence values are uniformly distributed, as discussed in^54^.

It must be pointed out that degeneration is a significant challenge in discrete chaotic maps used for cryptographic applications. It occurs when chaotic systems lose their inherent randomness due to limitations of finite precision in digital implementations, leading to periodic or fixed-point dynamics and degradation in key properties such as sensitivity to initial conditions. Solutions to this problem, such as improving computation precision, adopting hyperchaotic maps, or employing multiple chaotic systems, have been proposed. However, increasing precision or combining multiple systems can incur higher computational costs and complexity in implementation, which may hinder their practicality. The 1-DEC map combines trigonometric and exponential functions to achieve highly nonlinear dynamics, making it less degenerate than traditional 1D maps. These dynamics ensure that even under finite precision, the map maintains its chaotic properties, including sensitivity to initial conditions and ergodicity. This is demonstrated in a comparative analysis with traditional 1D maps like the Chebyshev map, the logistic map, and other recent maps 1-DCP^55^, and 1-DSP^56^.

#### Bifurcation diagram

**Fig. 7:**
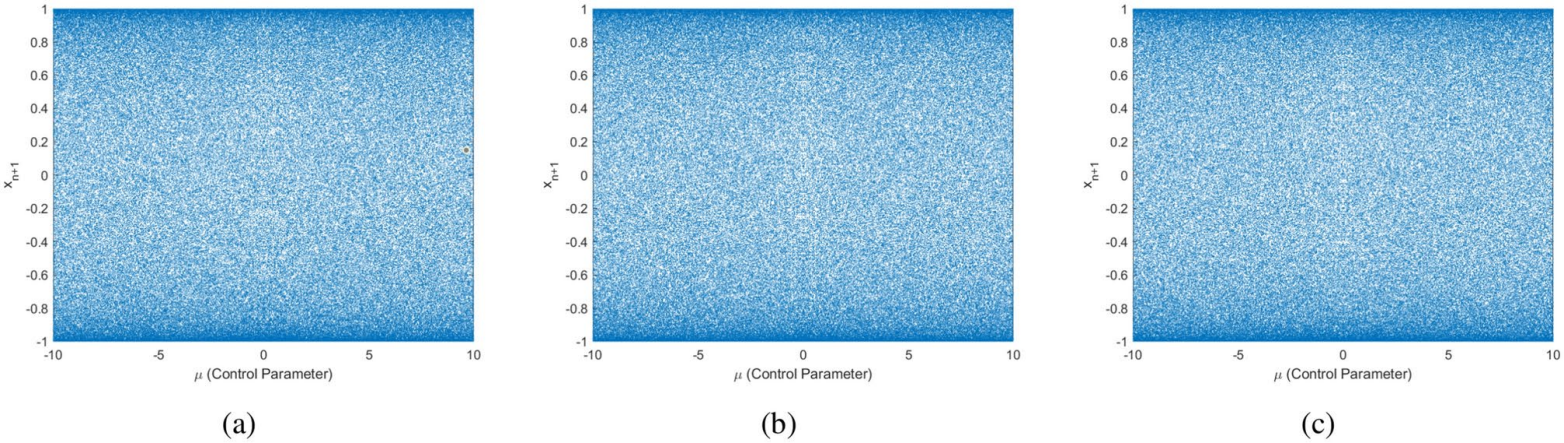
Bifurcation diagrams of the 1-DEC with varying initial conditions: (**a**) 0.25454, (**b**) 0.1, (**c**) 0.4.

**Fig. 8:**
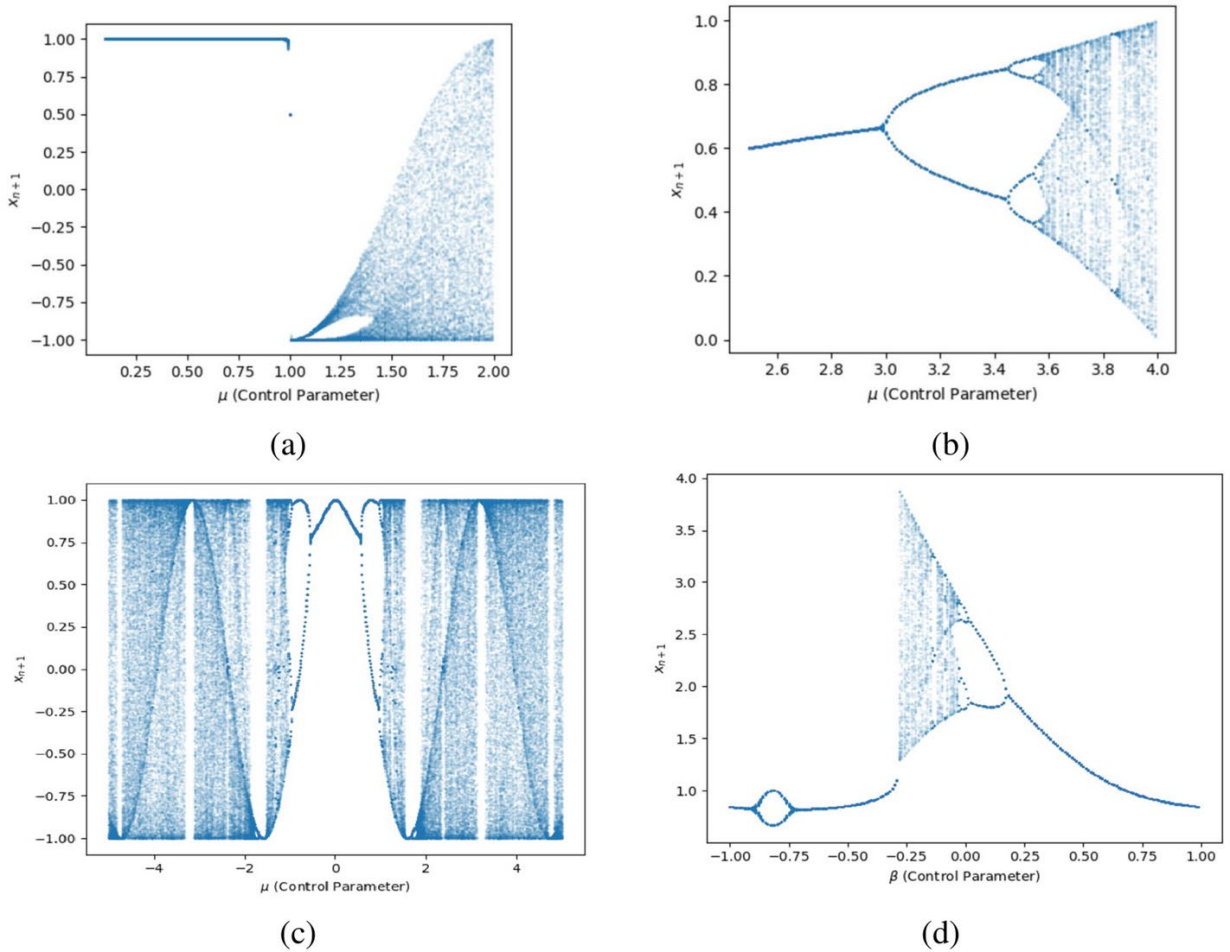
Bifurcation diagrams of (**a**) the Chebyshev map, (**b**) the logistic map, (**c**) 1-DCP map, and (**d**) 1-DSP map (control parameter $\alpha = 0.5$) with initial condition $0.25454$.

Considering the initial condition $y_1 = 0.25454$ (i.e., $n = 1$), iterating the 1-DEC for 150 cycles while altering the control parameter $\mu$ yields a sequence of length 150. To mitigate transient effects, the last 100 output values are chosen for 2D plot generation. The findings suggest that, regardless of fluctuations in the control parameter $\mu$, the sequence values generated by the 1-DEC show a uniform distribution across the entire space, spanning the complete range of mapping states. The same is true for the initial conditions $y_1 = 0.1$ and $y_1= 0.4$. Bifurcation diagrams are essential in both theoretical and applied dynamics, offering a clear visual view to explore and understand complex behaviors in nonlinear systems as parameters change. The bifurcation diagrams in Fig. 7 depict the behavior of the 1-DEC map under different initial conditions ($0.25454, 0.1, 0.4$) with the control parameter $\mu$ shown over the interval $[-10,10]$. These diagrams demonstrate that the sequence values produced by 1-DEC remain uniformly distributed across the range of mapping states, irrespective of fluctuations in the initial conditions or control parameter values within this range. This uniformity confirms the map’s strong chaotic properties and supports its utility in encryption, as it enhances resistance to predictability and plain-image attacks. In contrast, Fig. 8 highlights the limitations of simpler maps like the logistic and Chebyshev maps. The logistic map demonstrates chaotic behavior only within a narrow range of $\mu$ (close to $3.6$ to $4.0$) but converges to fixed points or periodic orbits outside this interval, reducing randomness. The Chebyshev map fails to exhibit chaos for $\mu < 1$, limiting its applicability. In a similar fashion, the 1-DCP and 1-DCP maps are also not consistently chaotic for all intervals of control parameters. The comparisons underscore the superiority of the 1-DEC map, as its bifurcation diagrams consistently demonstrate uniformity and robust chaotic behavior across a broader range of parameters and initial conditions.

#### Lyapunov Exponent

**Fig. 9:**
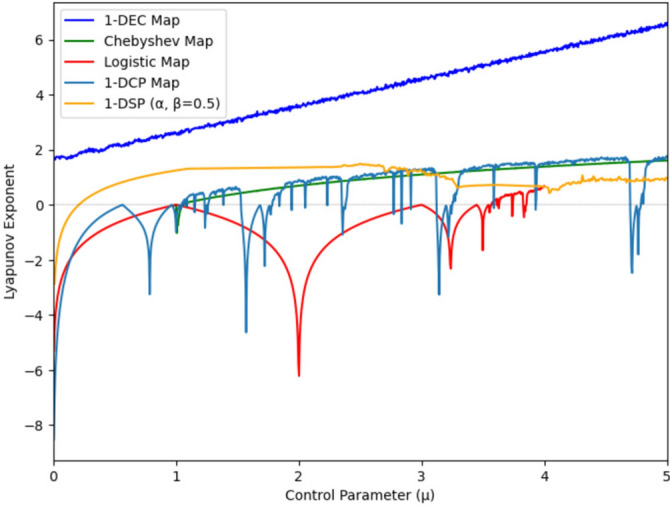
Lyapunov exponent analysis and comparison.

The Lyapunov Exponent (LE) quantifies the average rate of divergence or convergence of two infinitesimally close trajectories in the state space of a chaotic map, determining its sensitivity to initial conditions. It is mathematically expressed as:

$$\begin{aligned} \lambda = \lim _{n \rightarrow \infty } \frac{1}{n} \sum _{i=1}^n \ln \left| f'(x_i) \right| , \end{aligned}$$

where $f'(x_i)$ is the derivative of the map at the $i$-th iteration. A positive LE ($\lambda> 0$) across a wide range of control parameters indicates strong chaotic behavior and resilience against degeneration. The 1-DEC map’s superior LE ($\lambda> 0$ across its parameter range) confirms its robust chaotic behavior, as trajectories diverge exponentially even in discrete implementations as seen in comparative analyses with the other maps in Fig. 9.

The key advantage of employing the 1-DEC map lies in its straightforward hardware implementation. While higher-dimensional maps^57–60^ can offer increased complexity, they demand a greater number of logic gates, are often computationally expensive and pose challenges in precision management, as errors propagate more rapidly in the multi-dimensional system. One-dimensional maps provide a more efficient and less complex alternative. The proposed encryption algorithm uses a 1-DEC map that produces satisfactory results and is immune to plain-image attacks.

### VPD

To address the limitations of traditional DNA coding, a novel solution is the VPD encoding scheme published in^61^. This innovative approach creates 40320-rules for XORing with eight virtual planets: Uranus (Ur), Mars (Ma), Venus (Ve), Neptune (Ne), Jupiter (Ju), Earth (Ea), Saturn (Sa), and Mercury (Me). Each of these planets is uniquely identified by a 3-bit binary number in Table 2, allowing eight distinctive combinations. The VPD process involves applying the XOR operation, precisely a bitwise-XOR operation, among different planets. For instance, considering Venus (Ve) and Earth (Ea), which are represented as 010 and 101 in binary, the XOR operation yields 111, corresponding to the planet Mercury (Me). This standardized approach introduces a creative and systematic method for encoding binary information via a virtual solar system. The proposed approach uses Rule-37241 from^61^, which is listed in Table 3 as follows:

**Table 2:** Planets bitwise representation.

Planets	Ur	Ma	Ve	Ne	Ju	Ea	Sa	Me
Bit representation	000	001	010	011	100	101	110	111

**Table 3:** A comprehensive table illustrating the XOR operation between each virtual planet, utilizing Rule-37241, is shown in Table 2.

$\oplus$	**Ur**	**Ma**	**Ve**	**Ne**	**Ju**	**Ea**	**Sa**	**Me**
**Ur**	Ur	Ma	Ve	Ne	Ju	Ea	Sa	Me
**Ma**	Ma	Ur	Ne	Ve	Ea	Ju	Me	Sa
**Ve**	Ve	Ne	Ur	Ma	Sa	Me	Ju	Ea
**Ne**	Ne	Ve	Ma	Ur	Me	Sa	Ea	Ju
**Ju**	Ju	Ea	Sa	Me	Ur	Ma	Ve	Ne
**Ea**	Ea	Ju	Me	Sa	Ma	Ur	Ne	Ve
**Sa**	Sa	Me	Ju	Ea	Ve	Ne	Ur	Ma
**Me**	Me	Sa	Ea	Ju	Ne	Ve	Ma	Ur

The motivation for utilizing the 1-DEC and VPD domains in the proposed encryption algorithm is to achieve heightened key sensitivity and increased randomness, thereby bolstering the algorithm’s resilience against cryptographic attacks, particularly side-channel attacks, and ease of implementation on hardware (IoT devices).

## Key generation procedure

This section discusses the key generation procedure in detail, presents the randomness test results, and provides the corresponding pseudocode. The keys generated by the proposed method are verified and added through the NIST SP 800-22 statistical test suite to ensure their randomness. We use DCGAN to generate a random, fake-color image for key generation. The generated image is similar to the original image but can have very different RGB values. The base data set used for DCGAN training, which includes more than 10,000 images of skin cancer, is sourced from Kaggle (https://www.kaggle.com/code/jnegrini/ham10000-analysis-and-model-comparison). The architecture of the DCGAN involves a generator and discriminator, as explained in “DCGAN”. The training process requires multiple iterations to optimize the generator and discriminator networks until the generator can produce realistic synthetic images that closely mimic the distribution of the training data. The trained generator is ready for real-time key generation without additional computational overhead. We also used the time-stamp format: DD-MM-YYYY HH:MM:SS (Day-Month-Year Hour:Minute:Second) along with nonce during the key generation process along with 1-DEC; the steps are as follows:

- **Step 1:** Read the DCGAN-generated image; separate it into its red, green, and blue channels; and calculate the sum of pixels for each channel. After calculating the sum of the pixel values for each color channel, the code constructs a string sum containing the sums of the red, green, and blue channels incremented by 1, converted to strings. Additionally, the sum string is concatenated with a timestamp and a nonce. This step essentially supports the key space if all the pixel values are zero.
- **Step 2:** Sum converted to 512 Hash through SHA-512.
- **Step 3:** We divide the number into eight parts, convert every part into a decimal of the 15 digits, and divide it by $10^{15}$.
- **Step 4:** The initial condition for the 1-DEC is obtained in Step 3, which iteratively generates eight sequences of data streams. These streams are used in the proposed encryption algorithm to perform shuffle and plane XOR. We consider them keys ($K_1, K_2,\dots , K_8$).

The key generation procedure is further explained through pseudocode, which is provided in Algorithm 1.

**Algorithm 1:**
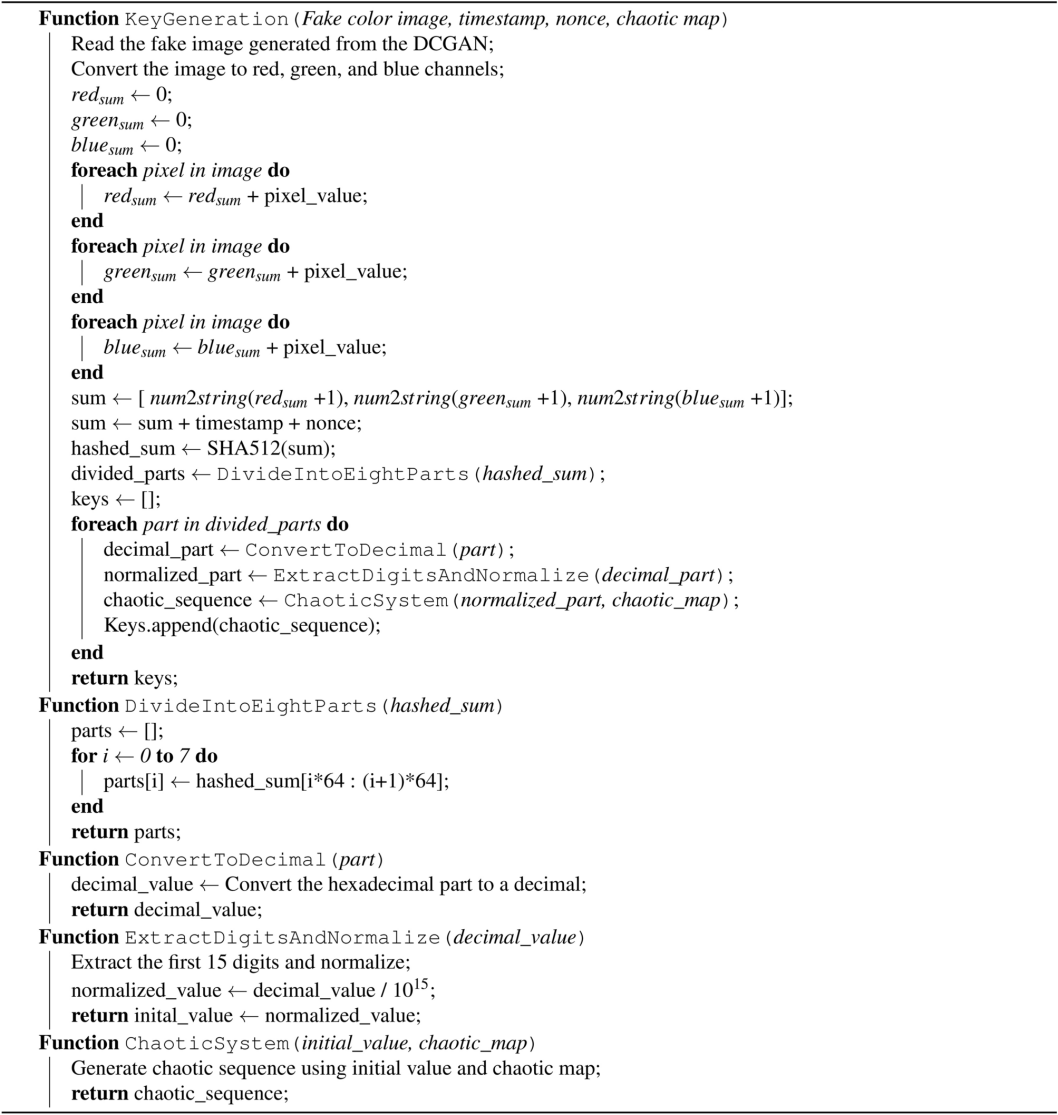
Key generation procedure

### Randomness results on keys ($K_1,K_2,\dots ,K_8$)

We generated 12,000,000 bits and divided them into ten batches, each containing 1,200,000 bits. These batches were analyzed using the NIST SP 800-22 statistical test suite, with the outcomes summarized in Table 4, where the P-value is abbreviated as P-val. The results in Table 4 demonstrate that the eight generated keys possess a high degree of randomness. Each P-value in the NIST test suite represents the probability that a perfect random number generator could produce a sequence exhibiting less randomness than the sequence being tested, based on the specific non-randomness criteria evaluated by the test. A P-value greater than 0.01 indicates that the sequence is considered random, while a P-value less than 0.01 suggests the sequence is non-random.

**Table 4:** NIST SP 800-22 Statistical test suite results on keys.

Statistical Tests	$K_1$ P-val	$K_2$ P-val	$K_3$ P-val	$K_4$ P-val	$K_5$ P-val	$K_6$ P-val	$K_7$ P-val	$K_8$ P-val	Range	Result
Frequency	0.739918	0.534146	0.534146	0.534146	0.122325	0.534146	0.911413	0.739918	0.122325–0.911413	$\checkmark$
Block Frequency	0.911413	0.739918	0.739918	0.911413	0.066882	0.739918	0.911413	0.213309	0.066882–0.911413	$\checkmark$
Cumulative Sums : Forward	0.911413	0.350485	0.350485	0.122325	0.534146	0.534146	0.350485	0.739918	0.122325–0.911413	$\checkmark$
Cumulative Sums : Reverse	0.739918	0.122325	0.066882	0.350485	0.213309	0.066882	0.122325	0.534146	0.066882–0.739918	$\checkmark$
Non Overlapping Template Matching	0.350485	0.213309	0.739918	0.739918	0.350485	0.739918	0.739918	0.066882	0.066882–0.739918	$\checkmark$
Overlapping Template Matching	0.911413	0.066882	0.911413	0.739918	0.739918	0.350485	0.739918	0.122325	0.066882–0.911413	$\checkmark$
Longest Run	0.066882	0.739918	0.534146	0.122325	0.122325	0.911413	0.066882	0.035174	0.035174–0.911413	$\checkmark$
Fast Fourier Transform	0.911413	0.350485	0.350485	0.534146	0.350485	0.911413	0.350485	0.213309	0.213309–0.911413	$\checkmark$
Matrix Rank	0.739918	0.534146	0.739918	0.911413	0.350485	0.350485	0.534146	0.911413	0.350485–0.911413	$\checkmark$
Serial 1	0.066882	0.534146	0.350485	0.534146	0.213309	0.534146	0.911413	0.739918	0.066882–0.911413	$\checkmark$
Serial 2	0.534146	0.350485	0.534146	0.534146	0.739918	0.534146	0.739918	0.017912	0.017912–0.739918	$\checkmark$
Universal	0.739918	0.534146	0.911413	0.350485	0.534146	0.739918	0.911413	0.350485	0.350485–0.911413	$\checkmark$
Approximate Entropy	0.213309	0.534146	0.911413	0.911413	0.213309	0.350485	0.534146	0.122325	0.122325–0.911413	$\checkmark$
Linear Complexity	0.739918	0.739918	0.534146	0.739918	0.911413	0.739918	0.991468	0.213309	0.213309–0.911413	$\checkmark$

## Proposed encryption and decryption process

In this section, we discuss the proposed encryption and decryption algorithms in more detail, along with pseudocodes providing the detailed explanation necessary for implementation.

### Proposed encryption process

We begin our encryption process by taking the user image, which we further need to divide into its three principal components, namely, the red, green, and blue channels. The steps are as follows:

- **Step 1: VPD ** Three RGB image channels are used. Each channel is converted into a binary bitstream. These three bitstreams are then concatenated into a single-row form.
  1. ** Generating sequence** $S_1$: A sequence called $S_1$ is constructed such that the first term of $S_1$ consists of the first three bits from the concatenated bitstream. The second term of $S_1$ includes the next three bits from the bitstream. This pattern is continued, where each subsequent $S_1$ term corresponds to the next set of 3 bits from the bitstream.
  2. **Generating sequence** $S_2$: Now, create a new sequence called $S_2$. The first term of $S_2$ remains the same as that of $S_1$. For the subsequent terms of $S_2$, the $i^{th}$ term of $S_2$ is obtained by applying the XOR operation between the $i^{th}$ term of $S_1$ and the $(i-1)^{th}$ term of $S_2$. In other words, $(S_2[i] = S_2[i-1]\oplus S_1[i])$ for $i\ge$2. After that, the bitstream $S_2$ is divided into three equal parts, and the resulting three mixed bitstreams are converted into similar types of channels, such as red, green, and blue, and then are combined to obtain a partially encrypted image. This step is crucial, as the adversary can attack any channel to avoid a channel attack. Additionally, an adversary cannot bypass this step, even if they use an image with uniform pixel values.
- **Step 2: 3D pixel intershuffling** After that, one can use a new secure “3D pixel intershuffling” method. In this method, three iterators, *i*, *j*, and *k*, are used, which iterate over the image in a looping manner. Consider a pixel at position $(i, j, k)$ in the image, where one can replace this pixel with the pixel available at position ($K_1[i]$ mod 255, $K_2[i]$ mod 255, $K_3[i]$ mod 255). In this way, one can generate very secure and random 3D pixel intershuffling.
- **Step 3: Zigzag XORing** Zigzag XORing is applied after finishing Step 2. The process of zigzag XORing takes an image and a key $K_4$ as parameters. It begins by transforming the key by taking its absolute value, multiplying it by $10^{15}$, and then flooring the result to obtain an integer. Next, it applies a modulo operation with 256 to ensure that the key remains within the range of [0,256]. After preparing the key, the function retrieves the size s of the input image I. It then iterates through each pixel of the image in a triple nested loop, traversing through its width, height, and channels. At each pixel, it performs a bitwise XOR operation between the pixel value I(*i*, *j*, *k*) and the transformed key, storing the result in the corresponding location of the output image.
- **Step 4: Repeat Steps 1, 2, and 3** After zigzag XORing, repeat Step 1; Step 2 with keys $K_5, K_6,$ and $K_7$; Step 3 with key $K_4$.
- **Step 5: Planet with key** $K_8$ Then, at a time *t*, find the distance between the Sun and Uranus (Ur), Mars (Ma), Venus (Ve), Neptune (Ne), Jupiter (Ju), Earth (Ea), Saturn (Sa), and Mercury (Me). These eight distances are arranged in ascending order, and binary values between 000 and 111 are assigned. Now, iterate the image over three channels by converting it to an 8-bit binary, taking the three values at the *i* index, concatenating them, and assigning them to their corresponding planet name. Take a triplet of bits from the proposed key $K_8$ after scaling its absolute value by a factor of 256 and XOR with the binary counterpart of the corresponding planet name. After this, the bitstream is divided into three parts: the red, green, and blue channels. These binary values are converted back into the original pixel values and concatenated to obtain our final encrypted image.

### Proposed decryption process

In this section, we focus on the decryption process of encrypted images to obtain the original image.

- **Step 1: Reverse the planet with key** $K_8$ For each set of bits representing a planet name for each channel of the encrypted image, the XOR operation is performed with the corresponding bits from key $K_8$. The bitstream is divided into three parts for the red, green, and blue channels. These binary values are converted back into the original pixel values and concatenated.
- **Step 2: Reverse VPD** In this process, each color channel is transformed into a binary format. The binary streams from the red, green, and blue channels are combined into a single binary stream.
  1. Generate sequence $S_1$: Construct a sequence called $S_1$ such that the first term of $S_1$ consists of the first three bits of the bitstream, the second term consists of the next three bits, and this pattern continues throughout the bitstream.
  2. Generate sequence $S_2$: Generate another sequence $S_2$ as follows: $(S_2[i] = S_1[i]\oplus S_1[i-1])$ by taking $S_1[1]$ as the initial term. After that, the bitstream $S_2$ is divided into three parts: the red, green, and blue channels and the bitstream is converted into an image.
- **Step 3: Reverse zigzag XORing** Consider the key $K_4$, transform it like
  $$\begin{aligned} K'_4 = \left\lfloor \left| K_4\right| \times 10^{15} \right\rfloor \mod 256, \end{aligned}$$
  where the symbol ‘$\lfloor \quad \rfloor$’ is defined as the greatest integer of a function, and ‘$|\quad |$’ is defined as a modulus function. We take the size s of the input image I. It then iterates through each pixel of the image in a triple nested loop, traversing through its width, height, and channels. At each pixel, it performs a bitwise XOR operation between the pixel value I(*i*, *j*, *k*) and the transformed key $K'_4$, and the result is stored in the corresponding location of the output image.
- **Step 4: Reverse 3D pixel intershuffling** Iterate over each pixel location and reverse the shuffling process using the original values of $K_1[i]$, $K_2[j]$, and $K_3[k]$ to relocate each pixel to its original position.
- **Step 5: Reverse Steps 2, 3, and 4** Repeat Steps 2, 3, and 4, and again Step 2 to return the original image. The encryption process in more detail is described in algorithms 2 and 4. The decryption process is explained in detail via Algorithms 3 and 5.

**Algorithm 2:**
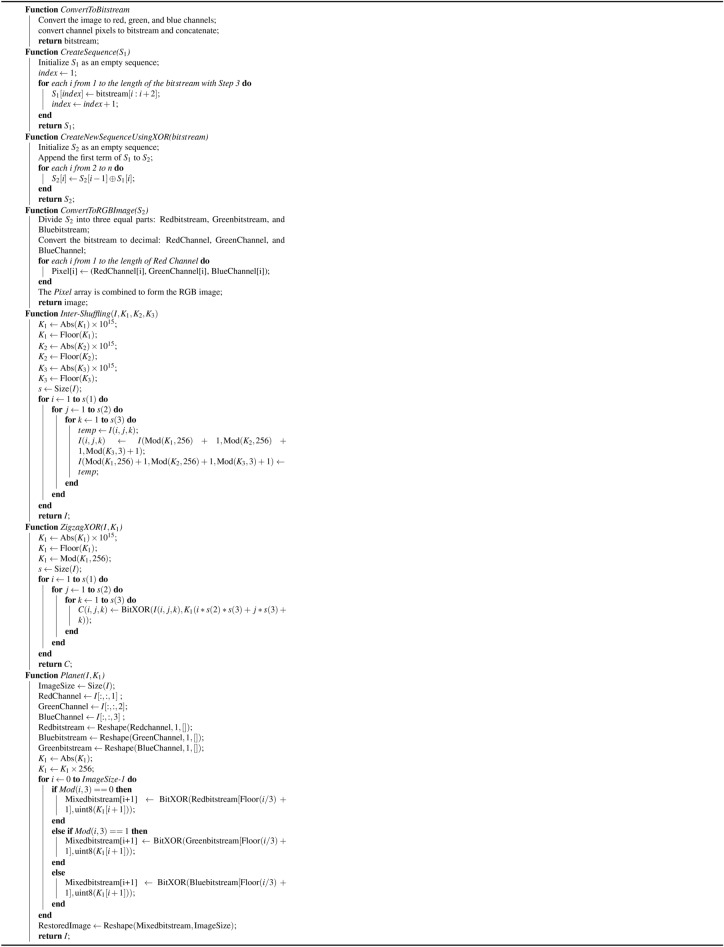
Functions used in the proposed encryption algorithm 4

**Algorithm 3:**
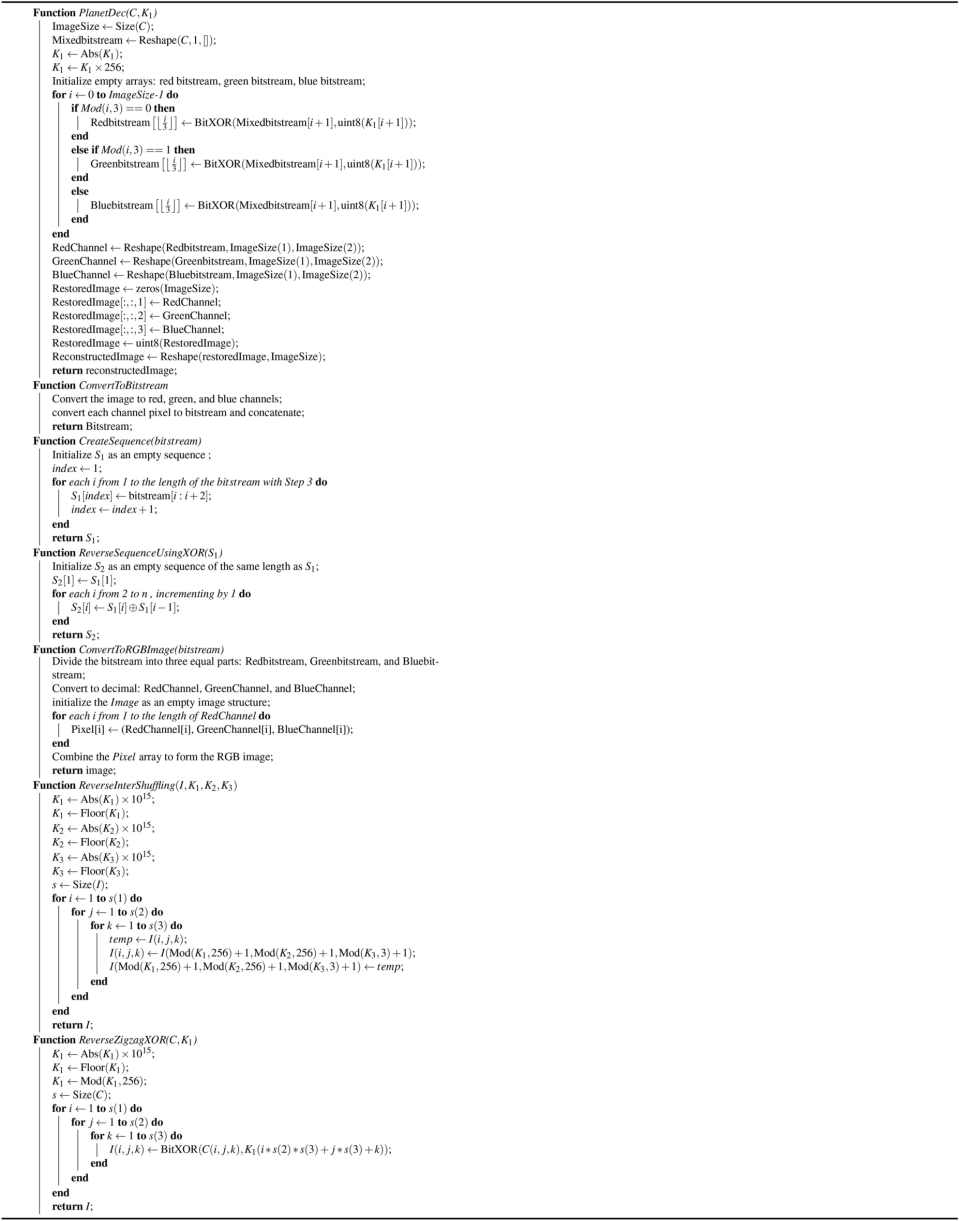
Reverse functions used in the proposed decryption algorithm 5

**Algorithm 4:**
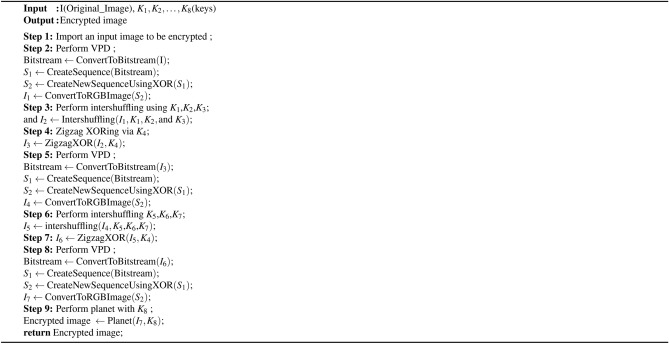
Proposed medical image encryption scheme

**Algorithm 5:**
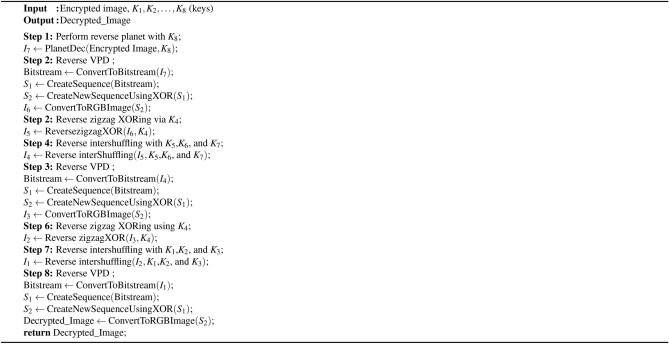
Proposed medical decryption scheme

### Encryption and decryption results

**Fig. 10:**
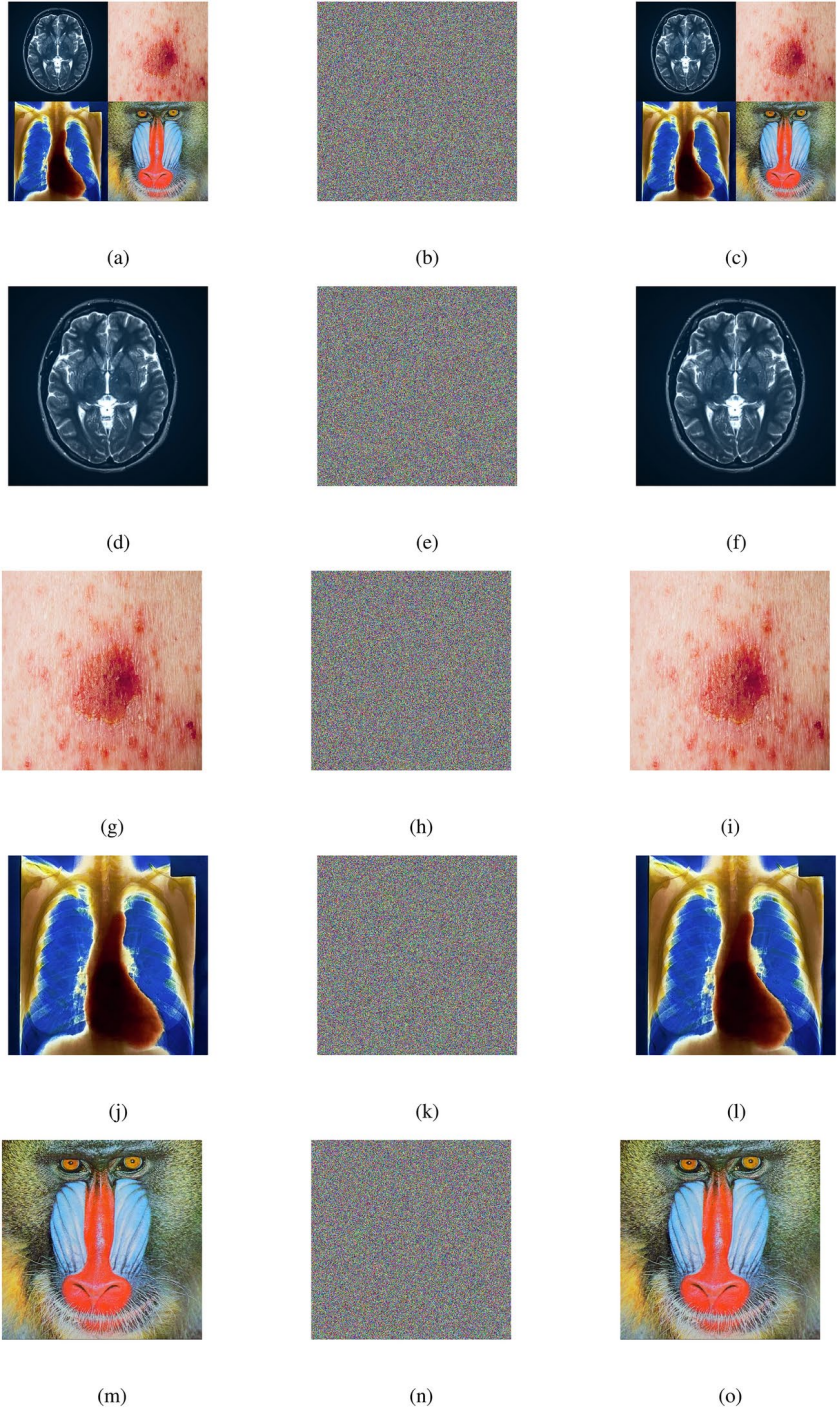
(**a**–**c**) Multiple images (original, encrypted, and decrypted), (**d**–**f**) brain MRI images (original, encrypted, and decrypted), (**g**–**i**) skin cancer images (original, encrypted, and decrypted), (**j**–**l**) chest X-ray images (original, encrypted, and decrypted), (**m**–**o**) Baboon images (original, encrypted, and decrypted).

The outputs of the proposed algorithm on medical images (multiple images, brain MRI images, skin cancer images, and chest X-ray images) and a test baboon image are shown in Fig. 10.

## Security analysis

In this section, a detailed performance and security analysis is presented to evaluate the robustness and effectiveness of the proposed encryption algorithm. The feasibility of the approach is demonstrated through simulation results, which highlight the algorithm’s ability to ensure randomness in the encrypted data. This randomness contributes significantly to the robustness of the encryption algorithm, even under adversarial attacks. Analyzing encrypted images involves examining the strength of their protective measures to ensure that they remain safe from unauthorized access or alterations.

### Time complexity

The time complexity of the encryption algorithm for an image of size $M \times N$, where $p=max(M,N)$, is determined by the individual complexities of its steps. The algorithm begins by converting the image into its RGB channels and subsequently into a concatenated bitstream. This step involves unpacking bits with a complexity of $O(M \times N \times 3)$ as there are three channels for RGB images. The next step constructs sequence $S_1$ by reshaping the bitstream, which is performed in $O(M \times N \times 3)$. The XOR operation to create $S_2$ uses a cumulative XOR computation, also taking $O(M \times N \times 3)$. The intershuffling step, which employs nested loops across the three dimensions of the image, has a complexity of $O(M \times N \times 3)$. The zigzag XOR operation iterates through all pixels, also contributing $O(M \times N \times 3)$. Final steps, such as repeated VPD operations and planet encryption (using a chaotic map), iterated over the entire bitstream and have complexities of $O(M \times N \times 3)$. Consequently, the overall encryption complexity is $O(M \times N)$. The decryption process mirrors the encryption steps, resulting in a similar time complexity of $O(M \times N)$. This analysis highlights the linear scalability of the proposed encryption and decryption processes with respect to the image size, ensuring computational efficiency for high-resolution images. Although encryption time is typically used to evaluate processing speed, various operating environments can affect it. The experimental simulations were conducted on an HPC server equipped with an AMD Ryzen 9 7950X 16-core processor with 4.5GHz, supported by 125 GB RAM. The proposed algorithm was implemented and tested using Python 3.11.7. The encryption and decryption execution times of the proposed algorithm for different-sized images are listed in Table 5.

**Table 5:** Encryption time (in seconds) for images of different sizes.

Image size	Encryption	Decryption
$64 \times 64$	0.29	0.27
$128 \times 128$	0.35	0.36
$256 \times 256$	0.63	0.60
$512 \times 512$	1.77	1.80

To ensure a fair comparison of the run time of the proposed algorithm with existing methods, we have evaluated it on a personal computer with specifications similar to those reported in other existing algorithms. Specifically, the tests were conducted on a system equipped with an 11th Gen Intel(R) Core(TM) i5-1135G7 processor running at 2.4 GHz and 16 GB of RAM. The results are summarized in Table 6.

**Table 6:** Average values of encryption time (in seconds) of image size 256 x 256 compared with existing methods.

Algorithms	Ref^62^	Ref^63^	Ref^64^	Ref^65^	**Proposed**
Execution time	5.77	6.39	10.82	1.72	5.31

### Key space analysis

The key space of a secure encryption algorithm must be larger than $2^{128}$to resist brute-force attacks^66^. The keyspace of the proposed algorithm is primarily determined by the precision of the eight initial values generated for chaotic sequence generation. These values are derived from the 512-bit output of SHA-512, which consolidates the entropy from the sum of pixel intensities of RGB channel sums of a DCGAN-generated image, a timestamp, and a nonce. While these inputs add randomness to the hashing process, the SHA-512 ensures a uniform 512-bit output, making the raw input complexity irrelevant to the keyspace. The 512-bit output is divided into eight parts, each contributing an initial value with a precision of $10^{-16}$. This precision leads to a total keyspace of approximately $(10^{16})^8 = 10^{128}$. Further, the encryption step involves planet XORing as provided in Table 3, which has 40320 options for choosing the planet XORing table. Hence, the resulting keyspace becomes $40320 \times 10^{128} \approx 2^{440}$ This ensures a vast keyspace, resistant to brute-force attacks, and comparable or superior to many existing encryption schemes, as shown in Table 7

**Table 7:** Key space of various image encryption algorithms.

Algorithms	Proposed	Ref^67^	Ref^68^	Ref^66^
Key space	$2^{440}$	$2^{297}$	$2^{351}$	$2^{345}$

### Histogram analysis

**Fig. 11:**
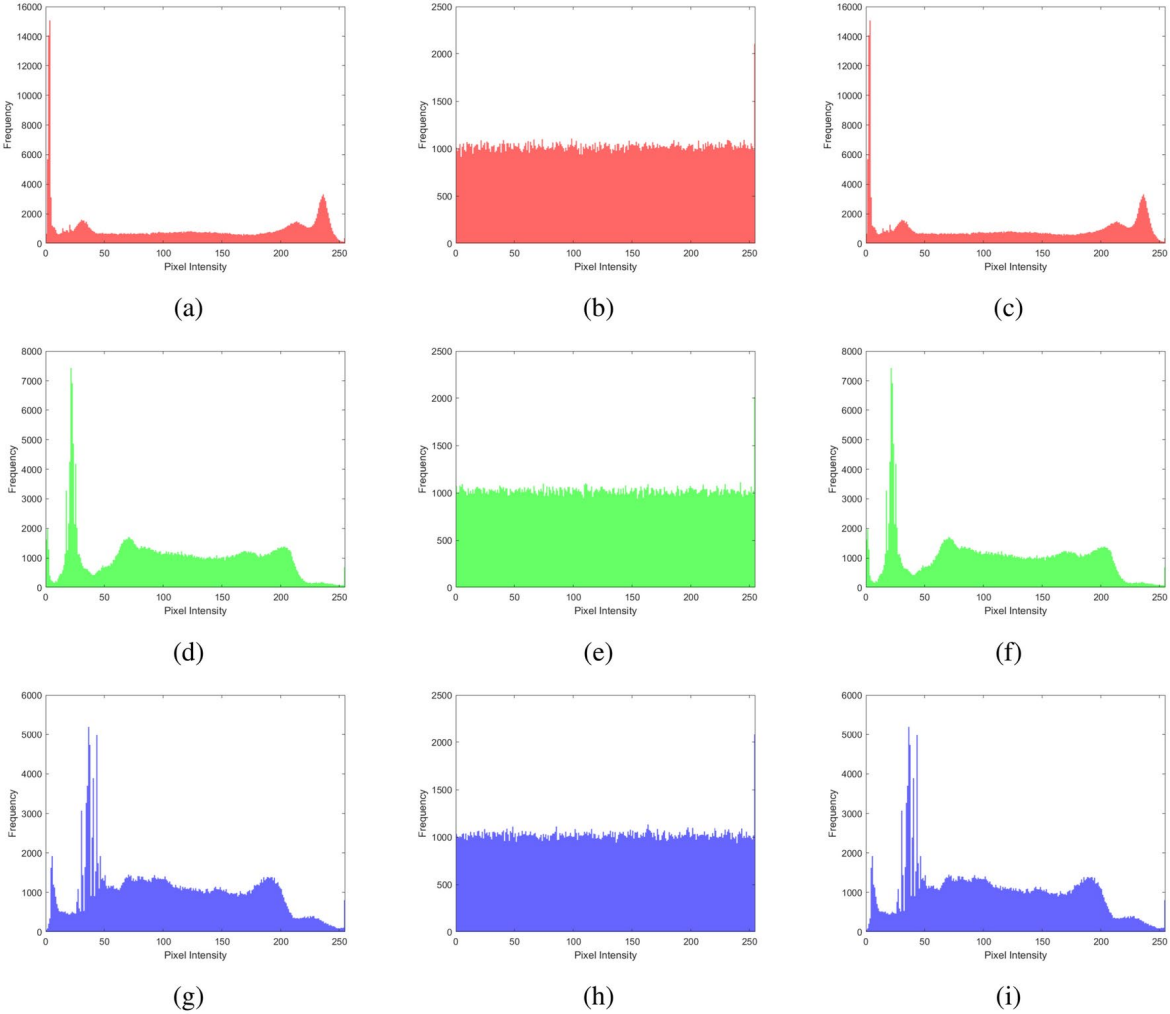
Histogram analysis of multiple images: (**a–c**) red channel (original, encrypted, and decrypted), (**d**–**f**) green channel (original, encrypted, and decrypted), and (**g**–**i**) blue channel (original, encrypted, and decrypted).

**Fig. 12:**
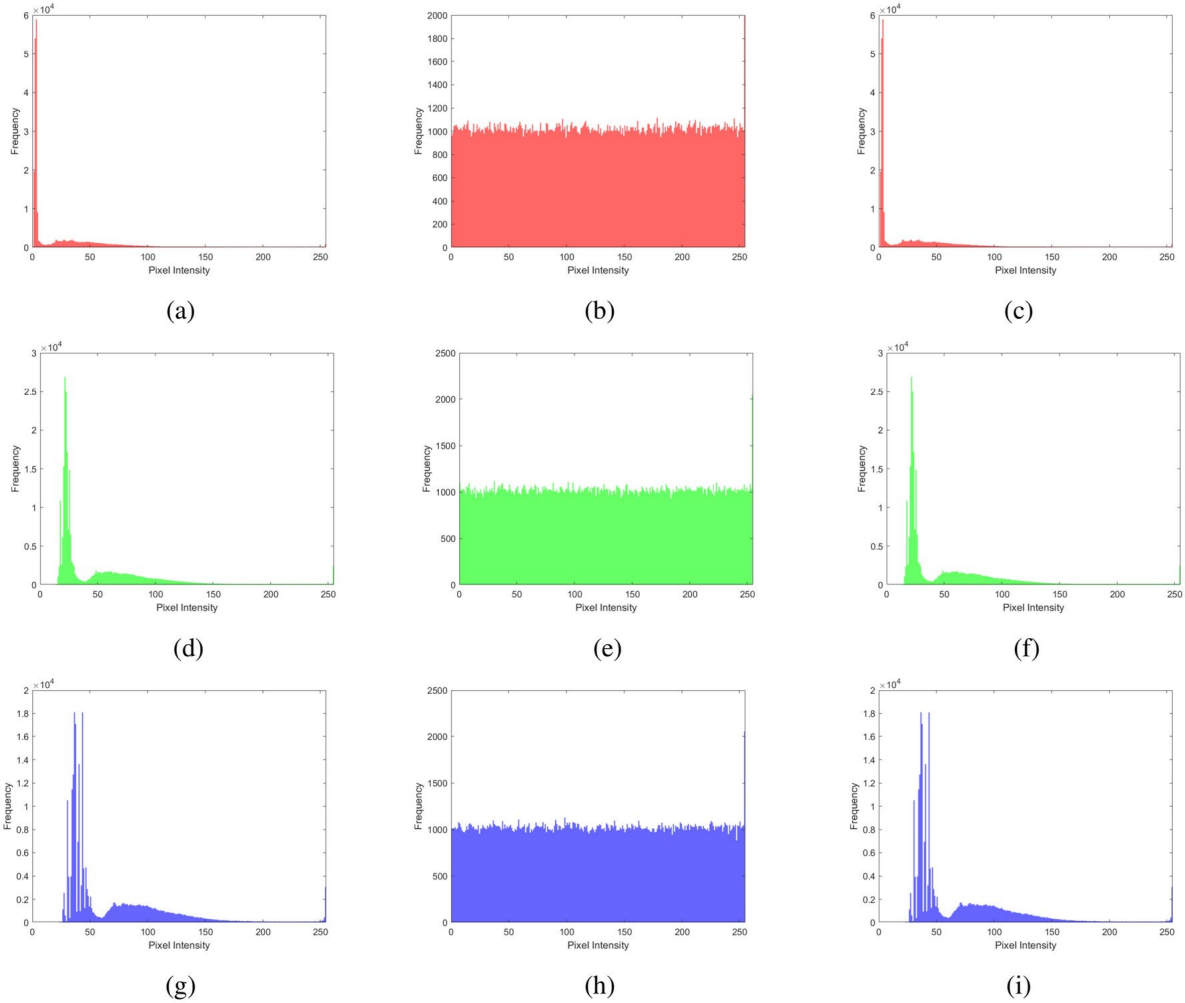
Histogram analysis of brain MRI image: (**a**–**c**) red channel (original, encrypted, and decrypted), (**d**–**f**) green channel (original, encrypted, and decrypted), and (**g**–**i**) blue channel (original, encrypted, and decrypted).

**Fig. 13:**
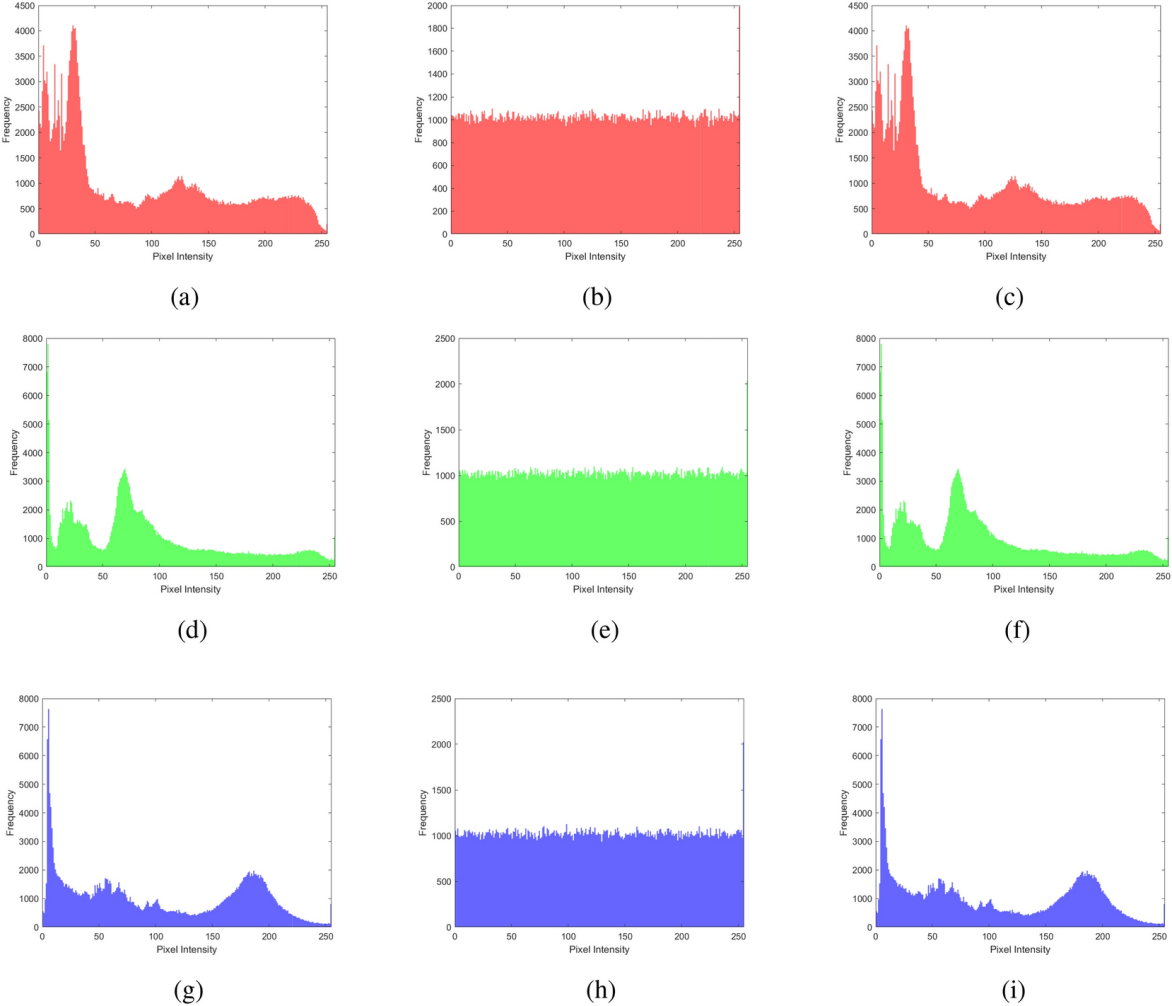
Histogram analysis of a chest X-ray image: (**a**) red channel (original, encrypted, and decrypted), (**d**) green channel (original, encrypted, and decrypted), and (**g**) blue channel (original, encrypted, and decrypted).

**Fig. 14:**
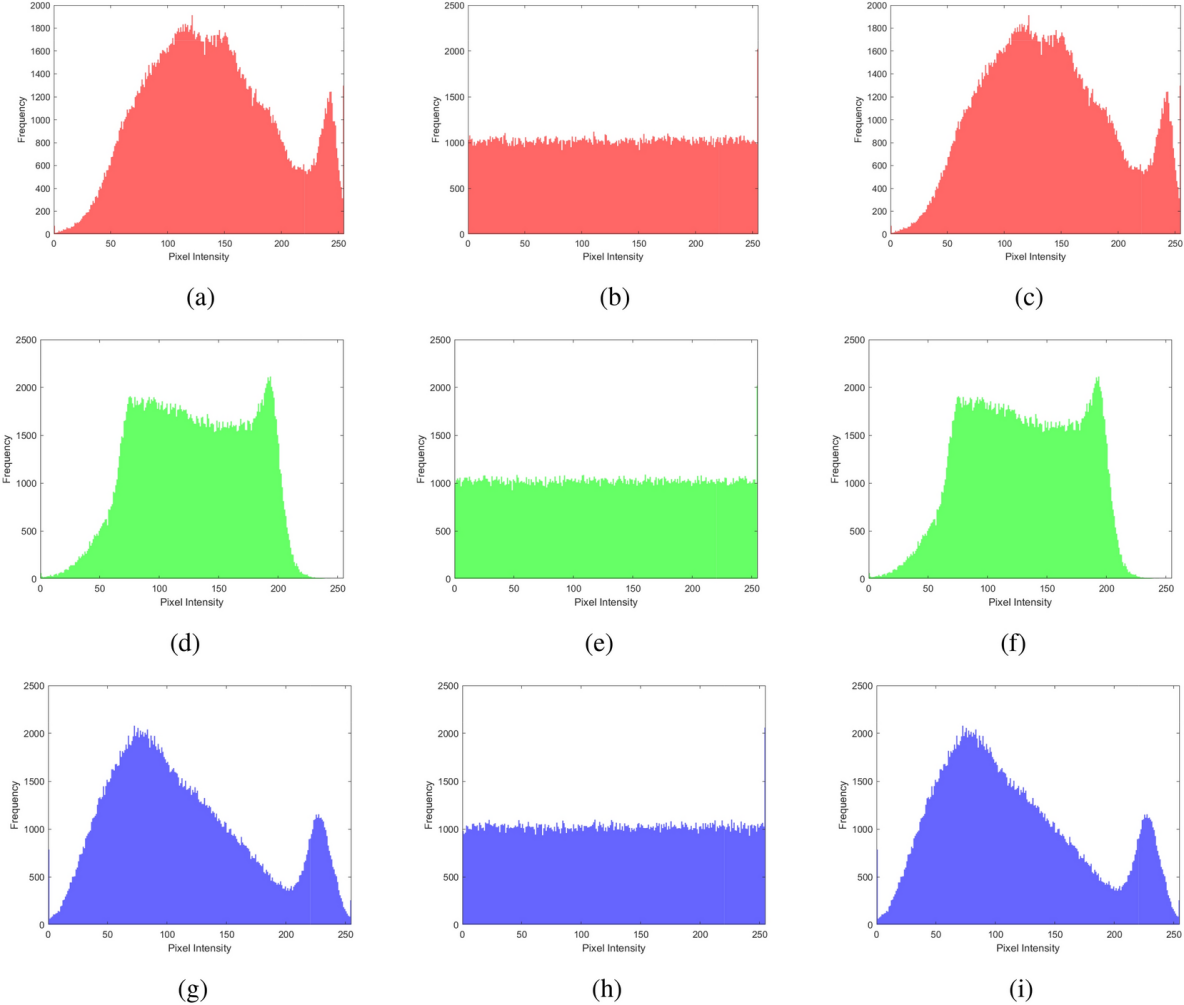
Histogram analysis of baboon image: (**a**) red channel (original, encrypted, and decrypted), (**d**) green channel (original, encrypted, and decrypted), and (**g**) blue channel (original, encrypted, and decrypted).

Histogram analysis provides a visual summary of the data distribution in a dataset. It shows the frequency or occurrence of different values within a range, helping to understand patterns such as peaks, gaps, or outliers. Displaying these frequencies as bars simplifies grasping the data’s central tendencies, spread, and overall shape, aiding in tasks like identifying common values, detecting anomalies, or making decisions on the basis of the data’s distribution. It is a graphical tool that provides an easy-to-understand overview of how values are distributed within a dataset. The histogram analysis of multiple images, brain MRI images, chest X-ray images, and baboon images are shown in Figs. 11, 12, 13, and 14. One can easily observe from the histogram of encrypted images in Figs. 11 (b, e, h), 12 (b, e, h), 13 (b, e, h), and 14 (b, e, h) show a constant level for each different image. Hence, we can conclude that the proposed algorithm is free from histogram attacks.

**Fig. 15:**
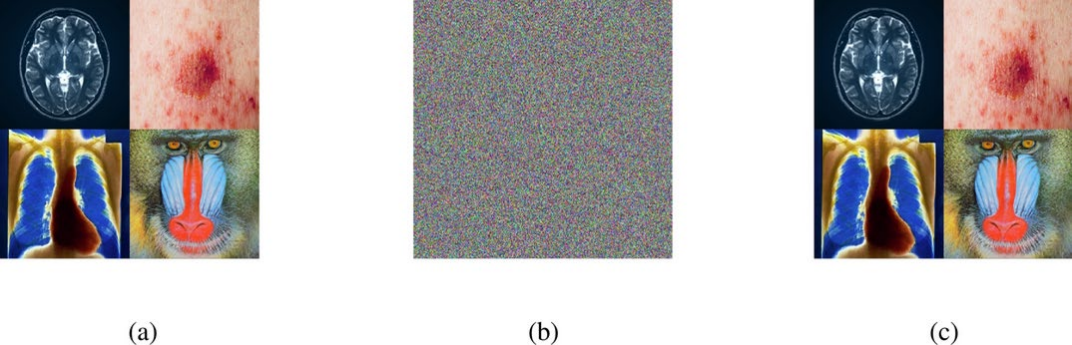
(**a**) Original multiple images, (**b**) Decrypted image with a small change in the initial condition of $K_4$, (**c**) Correctly decrypted image.

### Information entropy

One of the important measures is the entropy (*E*) of an image. It measures randomness or uncertainty in the source image, see reference for more details^69^. It is computed using the following formula provided in Eq. (2):

2 $$\begin{aligned} E(I) = -\sum _{i=0}^{N-1} p(i) \log _2(p(i)), \end{aligned}$$

where *I* and *N* denote the image and number of intensity levels in the image, and the term *p*(*i*) denotes the probability of occurrence of intensity level *i* in the image. To calculate *p*(*i*), one needs to count the frequency of each intensity level in the source image and divide it by the total number of pixels. The result of this formula represents the entropy in bits per pixel (bpp), indicating the average amount of information required to encode each pixel in the image. A higher entropy value suggests greater randomness or complexity within the image. The information entropy of the RGB components of different images (multiple images, brain MRI image, skin cancer image, chest X-ray image, baboon image) is shown in Table 8.

**Table 8:** Entropy for different encrypted images and their RGB components.

Test encrypted images	Entropy		
	R	G	B
Multiple images	7.9992	7.9993	7.9992
Brain MRI	7.9993	7.9993	7.9992
Skin cancer	7.9993	7.9992	7.9993
Chest X-ray	7.9994	7.9992	7.9993
Baboon	7.9993	7.9993	7.9993

### Key sensitivity

**Fig. 16:**
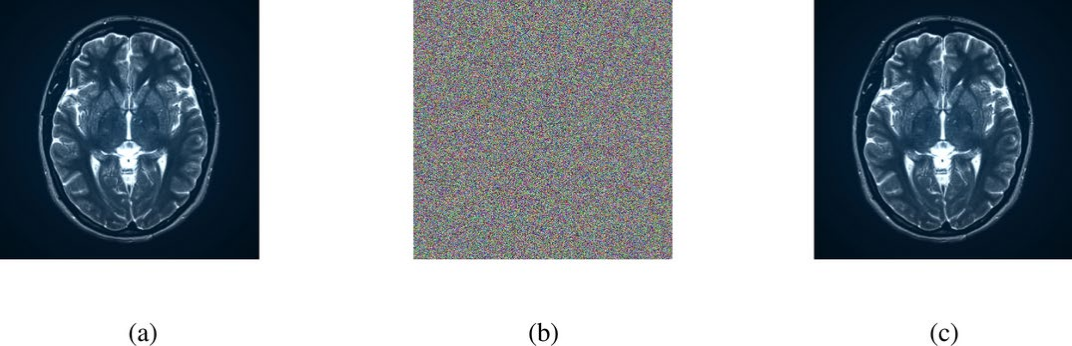
(**a**) Original brain MRI image, (**b**) Decrypted image with a small change in the initial condition of $K_4$, (**c**) Correctly decrypted image.

The proposed keys are sensitive to $10^{-16}$ decimal places. For instance, we demonstrate this statement via one of the keys, key 4 ($K_4$). The decryption process failed to yield the correct image when a perturbation of $10^{-16}$ was applied to the initial condition of $K_4$. Notably, a reduction in the perturbation to $10^{-17}$ resulted successfully ($\checkmark$) in recovering the original image during decryption. This observation highlights the algorithm’s sensitivity to minute variations in the key. The nuanced behavior underscores the significance of precise key management for ensuring the reliability and security of the cryptographic system. Thorough documentation remains imperative for comprehensive security assessments and the ongoing maintenance of system integrity. Decryption using a very marginal change, i.e., adding “$10^{-16}$” and “$10^{-17}$” to the initial condition of $K_4$ performed on the images (multiple images and brain MRI image) are shown in Figs. 15 and 16.

**Fig. 17:**
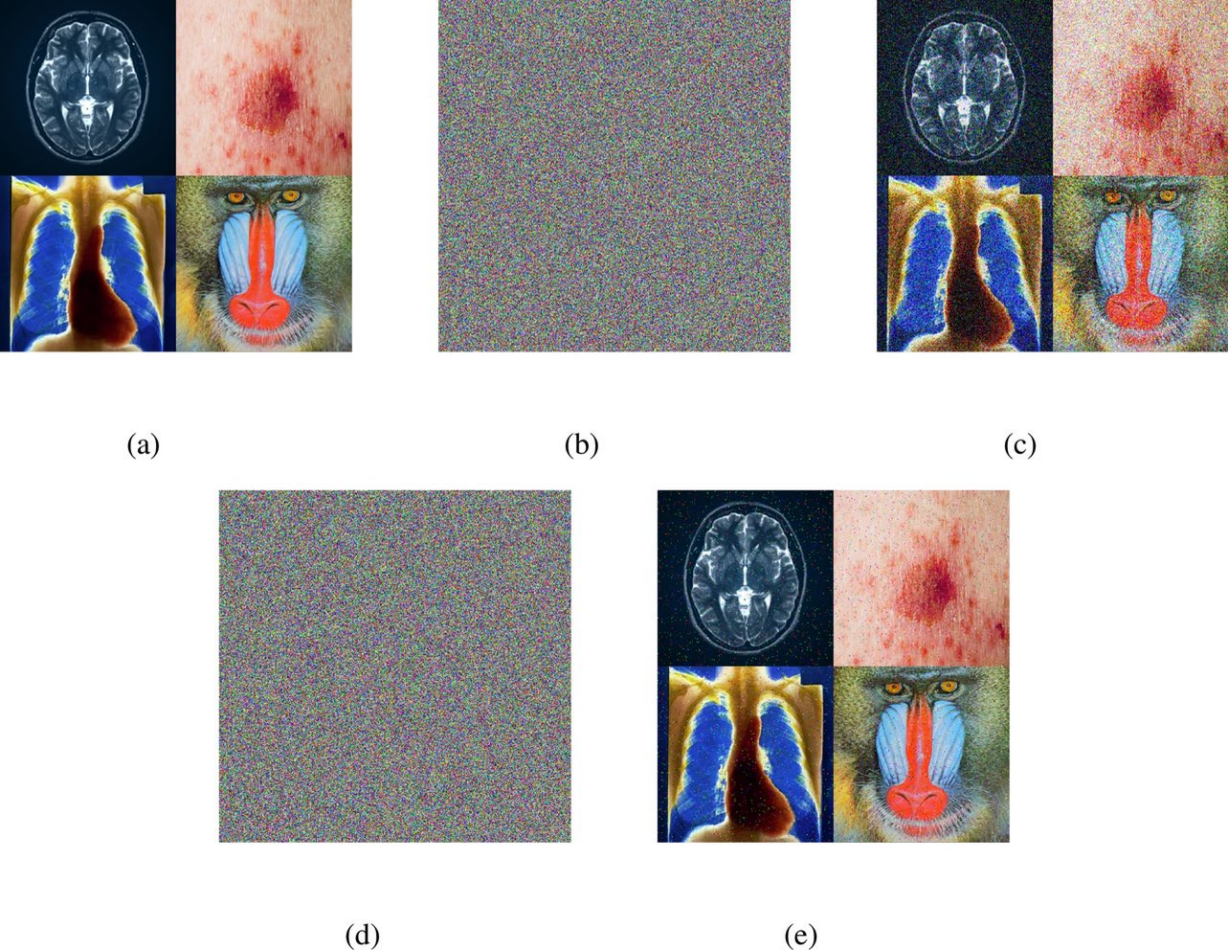
(**a**) Original multiple images: (**b**) encrypted multiple images with 0.05% Gaussian noise, (**c**) decrypted multiple images with 0.05% Gaussian noisy encrypted image, (**d**) encrypted multiple images with 0.02% salt and pepper noise, (**e**) decrypted multiple images with 0.02% salt and pepper noisy encrypted image.

### Add noise to the encrypted image

**Fig. 18:**
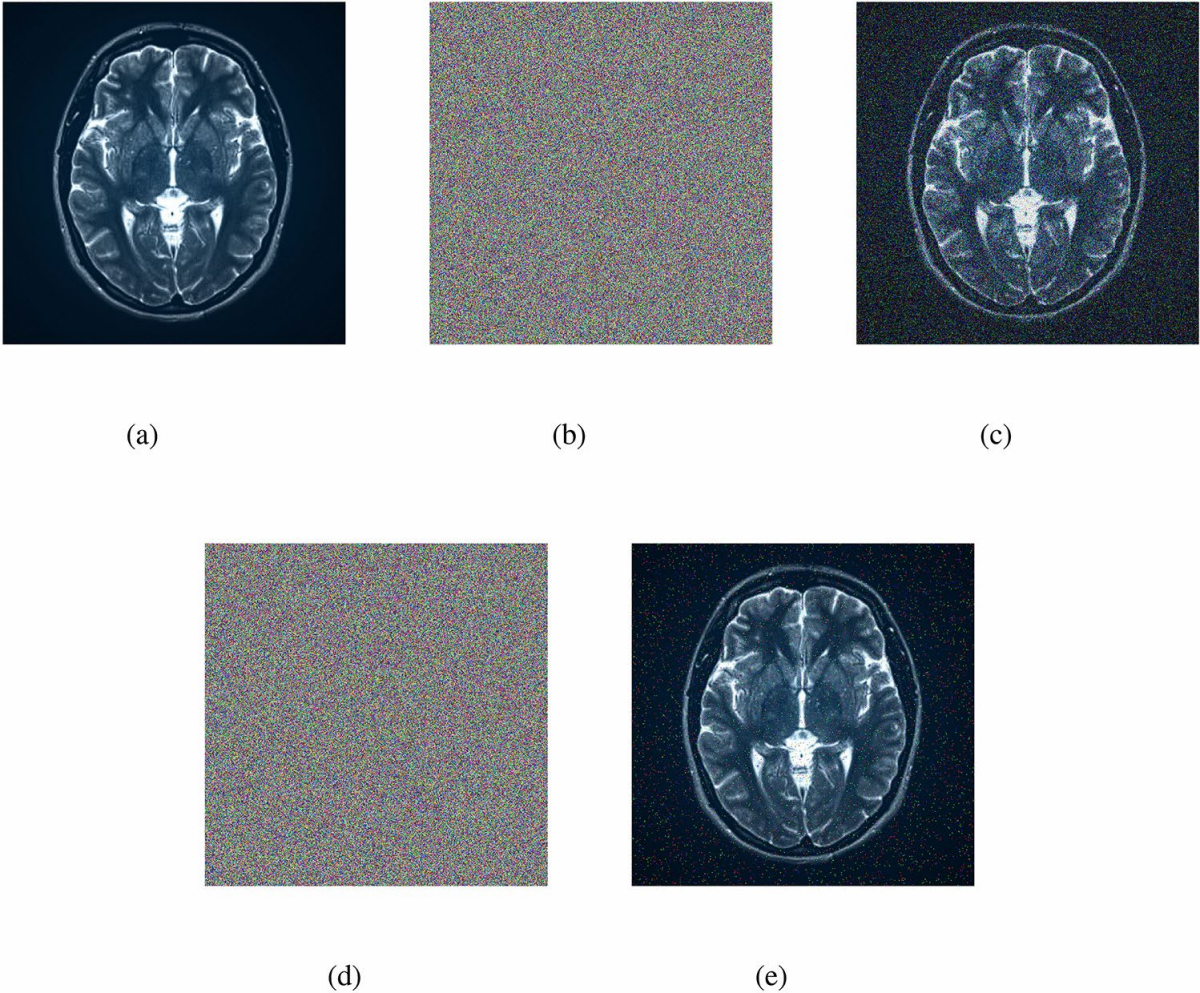
(**a**) Original brain MRI image, (**b**) encrypted brain MRI image with 0.05% Gaussian noise, (**c**) decrypted brain MRI image with 0.05% Gaussian noisy encrypted image, (**d**) encrypted brain MRI image with 0.02% salt & pepper noise, (**e**) decrypted brain MRI with 0.02% salt & pepper noisy encrypted image.

Adding noise to an encrypted image is a method to make the encrypted data more secure. It works by scrambling the encrypted image with random information (noise) to hide any patterns that might be present. Here, we use white Gaussian noise (0.05%) and salt & pepper noise (0.02%). When the image is decrypted, this added noise ensures that the final image is clear and accurate. This process helps protect sensitive images from certain attacks, increasing the resistance of the encrypted data to unwanted access or deciphering attempts. The encrypted images obtained by adding noise to the original images (multiple images, brain MRI image) and the decrypted images are shown in Figs. 17 and 18.

**Fig. 19:**
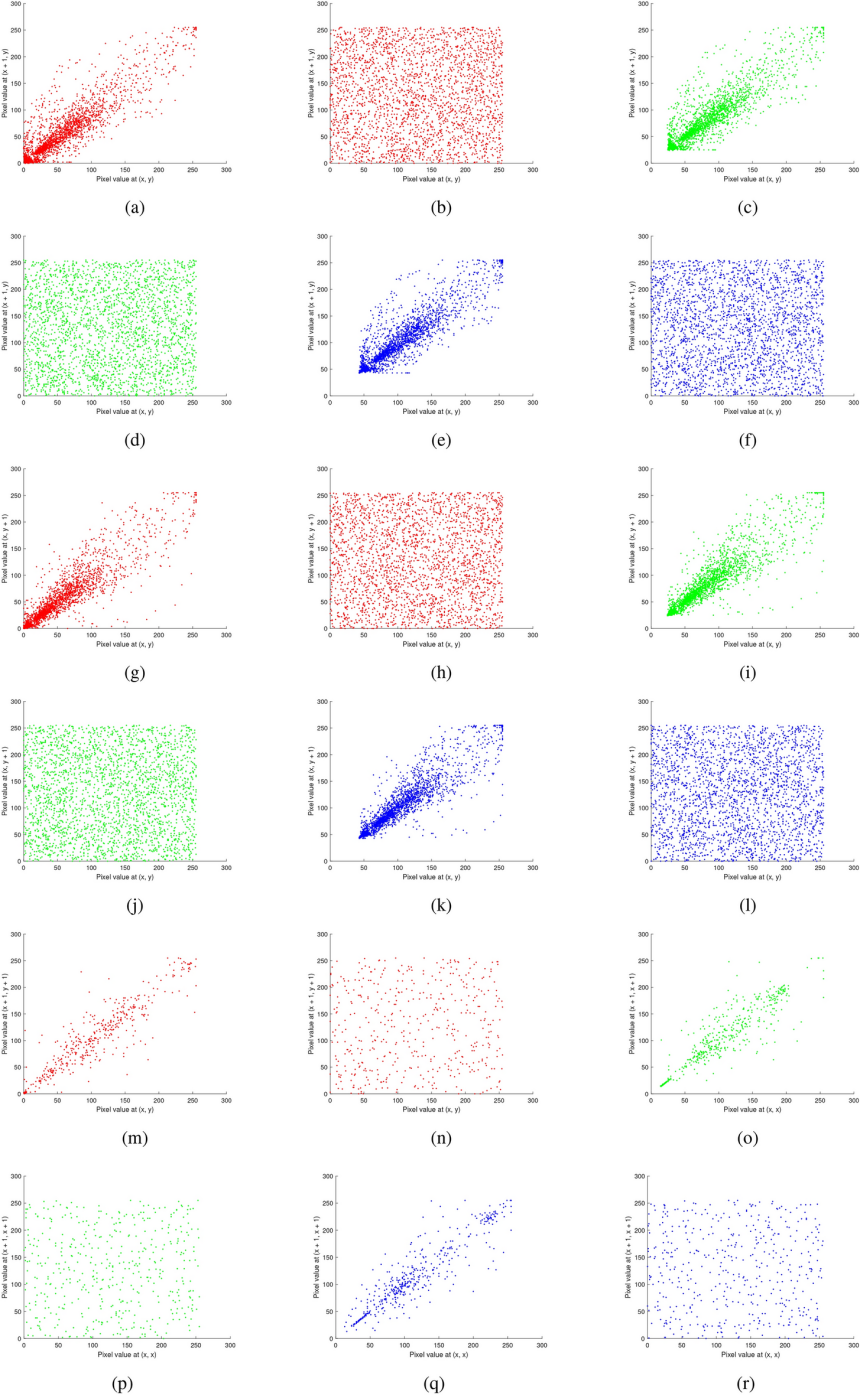
Correlation analysis of the multiple images before and after encryption: (**a**–**b**) red channel horizontal (original, encrypted), (**c**–**d**) green channel horizontal (original, encrypted), (**e**–**f**) blue channel horizontal (original, encrypted), (**g**–**h**) red channel vertical (original, encrypted), (**i**–**j**) green channel vertical (original, encrypted), (**k**–**l**) blue channel vertical (original, encrypted), (**m**-**n**) red channel diagonal (original, encrypted), (**o**–**p**) green channel diagonal (original, encrypted), and (**q**–**r**) blue channel diagonal (original, encrypted).

**Fig. 20:**
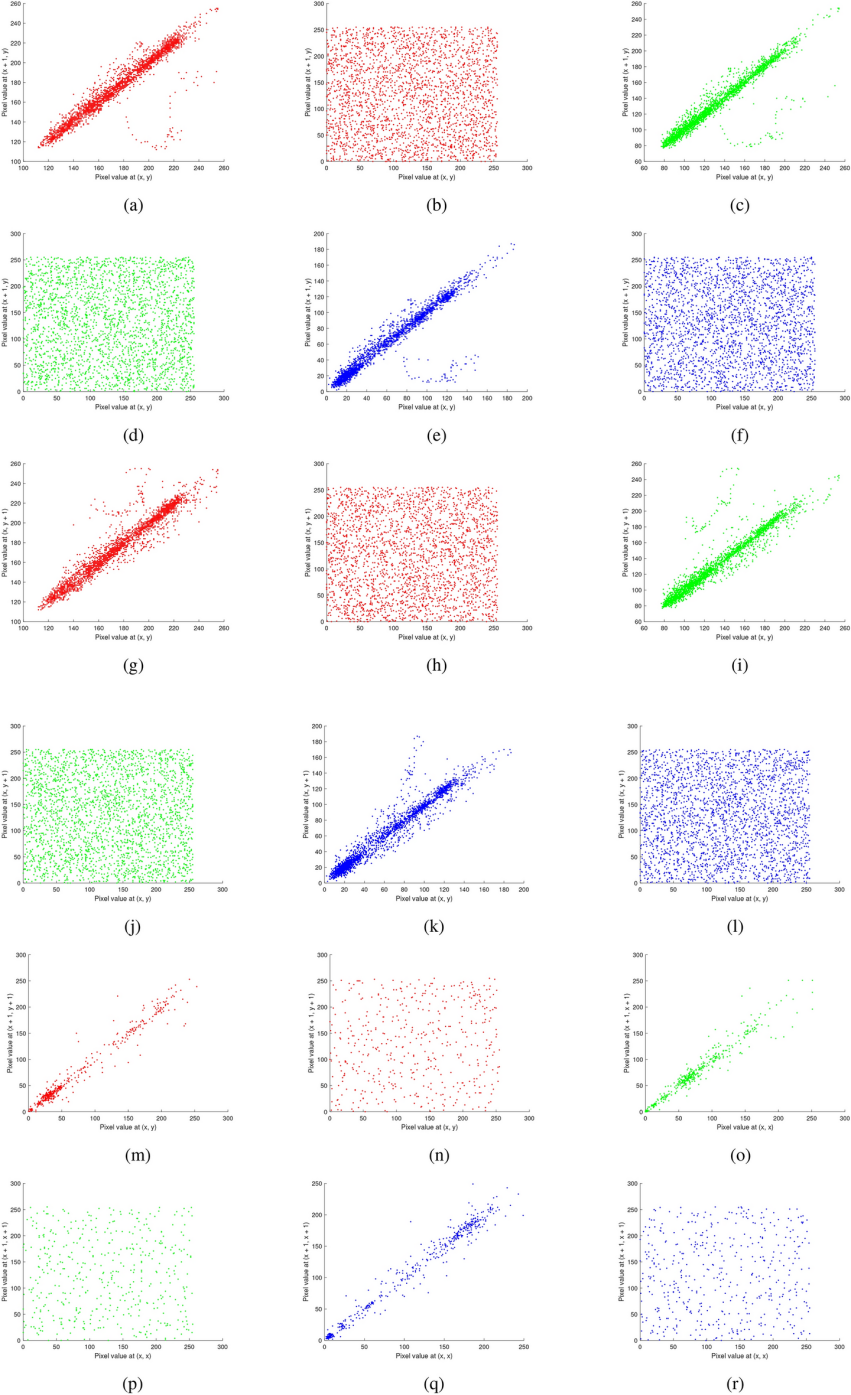
Correlation analysis of the chest X-ray image before and after encryption: (**a–b**) red channel horizontal (original, encrypted), (**c–d**) green channel horizontal(original, encrypted), (**e–f**) blue channel horizontal (original, encrypted), (**g–h**) red channel vertical (original, encrypted), (**i–j**) green channel vertical (original, encrypted), (**k–l**) blue channel vertical (original, encrypted), (**m–n**) red channel diagonal (original, encrypted), (**o**–**p**) green channel diagonal (original, encrypted), and (**q**–**r**) blue channel diagonal (original, encrypted).

### Correlation analysis

**Fig. 21:**
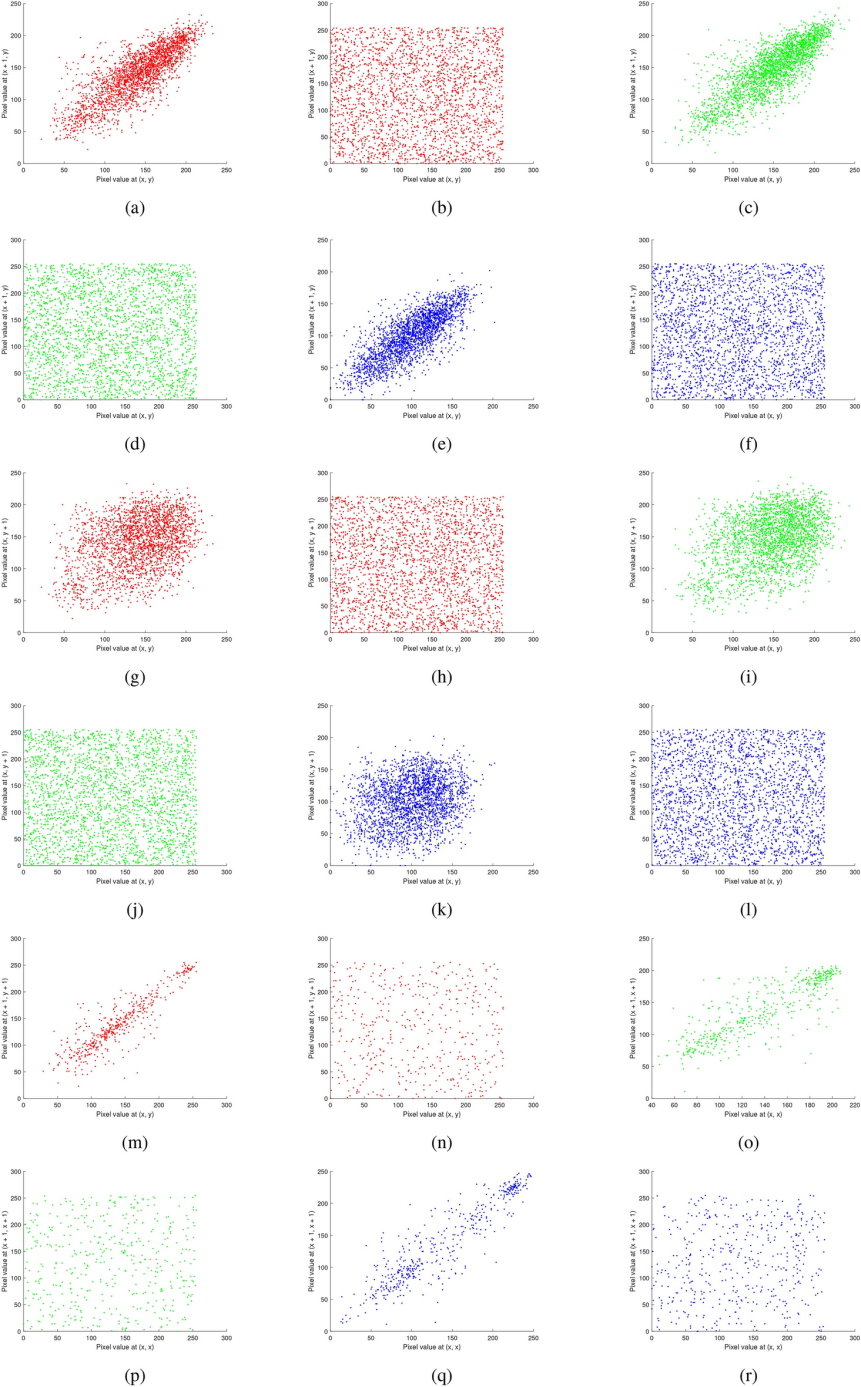
Correlation analysis of the baboon image before and after encryption: (**a**–**b**) red channel horizontal (original, encrypted), (**c**–**d**) green channel horizontal (original, encrypted), (**e**–**f**) blue channel horizontal (original, encrypted), (**g**–**h**) red channel vertical (original, encrypted), (**i**–**j**) green channel vertical (original, encrypted), (**k**–**l**) blue channel vertical (original, encrypted), (m-n) red channel diagonal (original, encrypted), (**o**–**p**) green channel diagonal (original, encrypted), and (**q**–**r**) blue channel diagonal (original, encrypted).

Table 9 displays the correlation coefficient values calculated using equation (3) for both the original and encrypted images for horizontal, vertical, and diagonal directions. The results (both before and after encryption) are plotted and shown in Figs. 19-21. The following formula is used to determine the correlation coefficient:

3 $$\begin{aligned} \left. \begin{array}{l} r_{uv} = \frac{\text {cov}(u, v)}{\sqrt{D_u D_v}}, \ \text {where} \ \text {cov}(u, v) = E[(u - E(u))(v - E(v))] \ \text {is the covariance of} \ u \ \text {and} \ v, \\ E(u) = \frac{1}{N} \sum _{i=1}^{N} u_i \ \text {is the expected value of} \ u, \\ \\ D_u = \frac{1}{N} \sum _{i=1}^{N} (u_i - E(u))^2 \quad \text {is the variance of u.}\\ \end{array} \right\} \end{aligned}$$

In this context, $u$ and $v$ denote the values of two adjacent pixels while $N$ represents the total number of samples. The analysis demonstrates that the proposed encryption scheme effectively diminishes the correlation between adjacent pixels in the encrypted images, enhancing security and data confidentiality. The results show that although the encryption process disrupts the original pixel correlations, these correlations are accurately restored during decryption.

**Table 9:** Correlation values of encrypted images for RGB components.

Testing encrypted image	Horizontal			Vertical			Diagonal		
	R	G	B	R	G	B	R	G	B
Multiple images	−0.0005	0.0093	−0.0549	0.0161	0.0051	0.0181	−0.0016	−0.0387	−0.0820
Brain MRI	0.0045	−0.0314	0.0114	0.0197	−0.0154	−0.0014	0.0918	0.0185	−0.0231
Skin cancer	0.0218	0.0026	0.0158	0.0041	−0.0304	−0.0110	−0.0272	−0.0241	−0.0184
Chest X-ray	0.0375	0.0035	−0.0128	0.0031	−0.0066	0.0124	0.0412	0.0833	−0.0345
Baboon	0.0128	0.0060	0.0036	0.0057	0.0171	−0.0166	−0.0783	−0.0079	0.1031

Correlation plots of original, encrypted, and decrypted images of different images are shown in Figs. 19, 20, and 21.

**Fig. 22:**
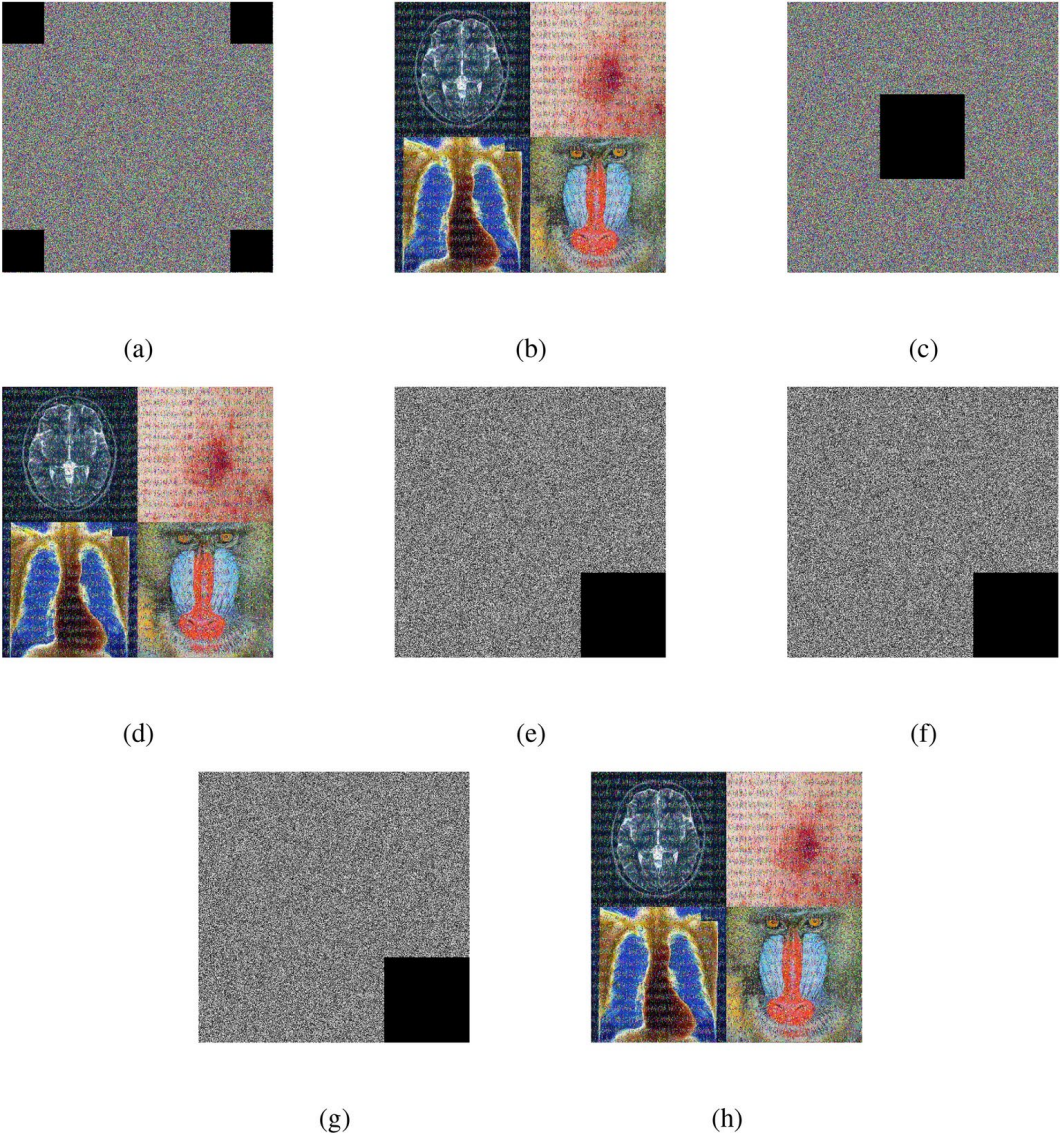
Multiple images: (**a**–**b**) four corner cropped (encrypted image, decrypted image), (**c–d**) center cropped (encrypted image, decrypted image), (**e**) red channel one corner cropped encrypted image, (**f**) green channel one corner cropped encrypted image, (**g**) blue channel one corner cropped encrypted image, and (**h**) one corner cropped decrypted image.

**Fig. 23:**
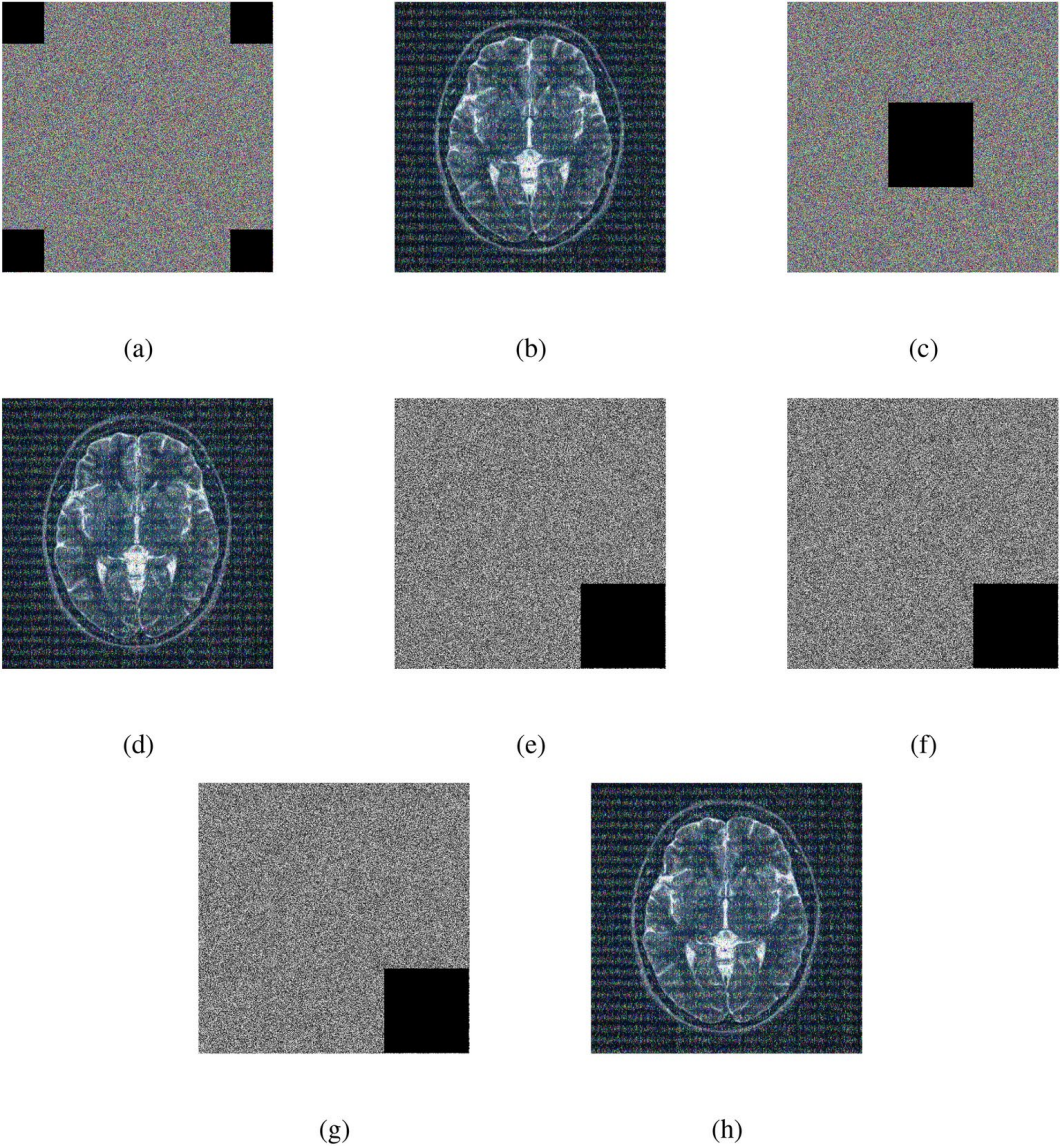
Brain MRI image: (**a–b**) four corner cropped (encrypted image, decrypted image), (**c**–**d**) center cropped (encrypted image, decrypted image), (**e**) red channel one corner cropped encrypted image, (**f**) green channel one corner cropped encrypted image, (**g**) blue channel one corner cropped encrypted image, and (**h**) one corner cropped decrypted image.

**Fig. 24:**
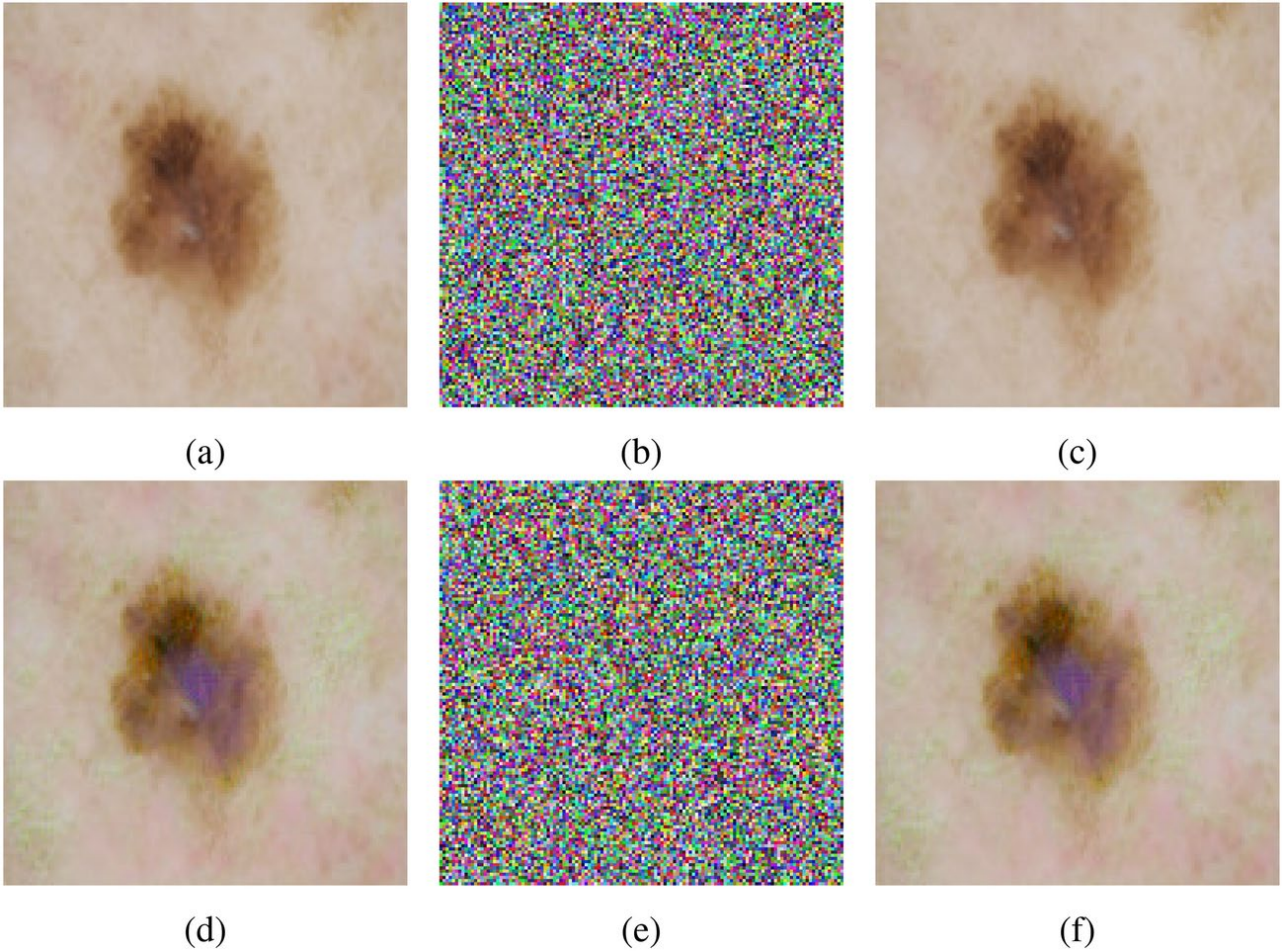
Adversarial attack using Skin cancer images (**a**–**c**) Original image encrypted and decrypted, (**d**–**f**) Adversarial image encrypted and decrypted.

**Fig. 25:**
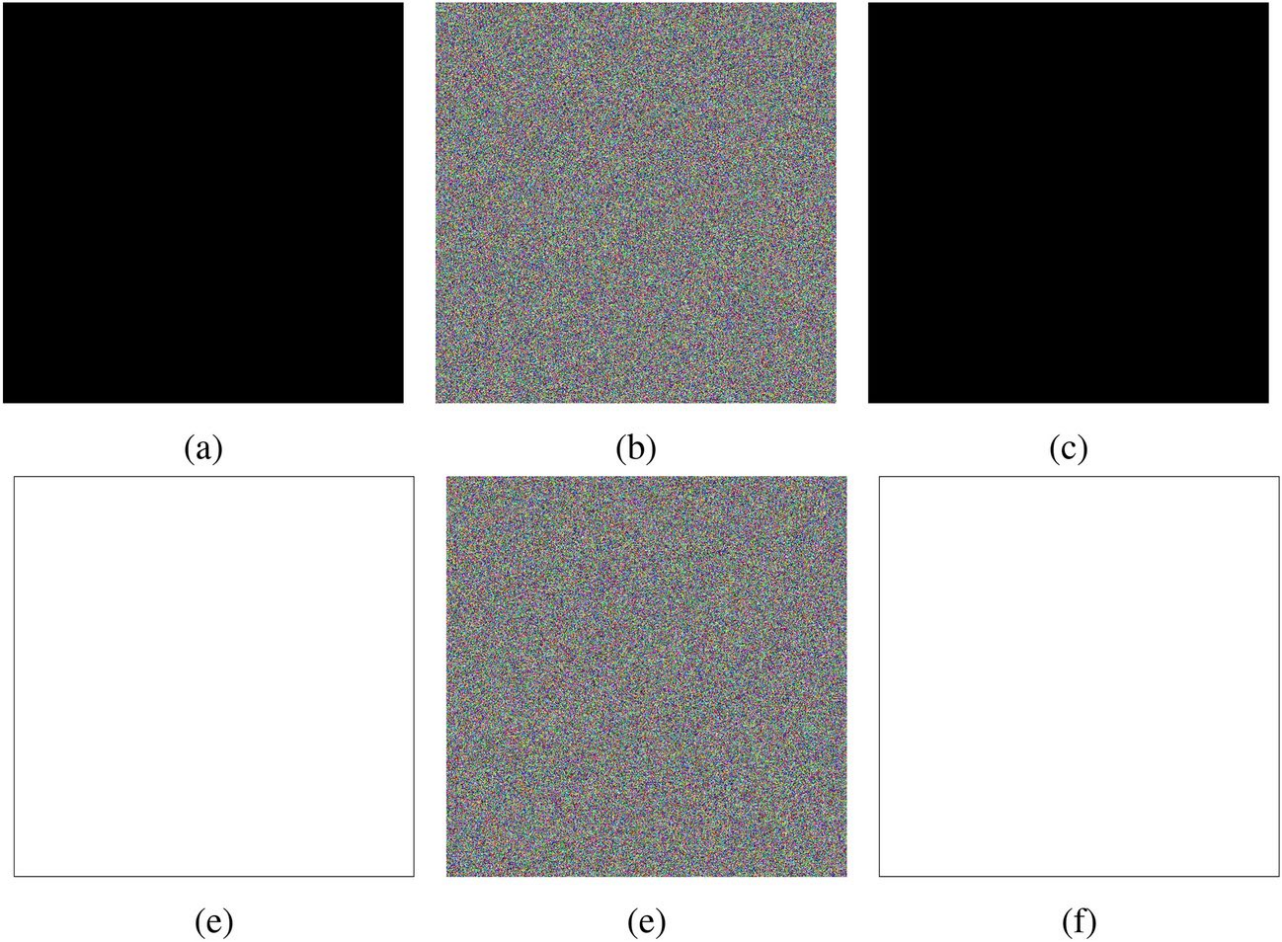
Plain, cipher, and decrypted images of all-black **(a–c**) and all-white (**d–f**) images.

### Robustness against cropping attacks

Protecting against cropping tricks means teaching programs to remain accurate even when some parts of an image are changed or removed. It shows the program many different image versions where parts are cut off. Figures 22 and 23 show cropping attacks for different images.

### Robustness against adversarial attacks

The encryption step effectively obfuscates adversarial patterns, safeguarding sensitive medical images during transmission or storage. By encrypting adversarial images generated using FGSM^70 ^and DeepFool^71^ methods, the algorithm disrupts the visibility of adversarial perturbations, rendering them inaccessible to potential attackers without the decryption key. Figure 24 comparing the original, adversarial, encrypted, and decrypted images demonstrates how the encryption process masks adversarial patterns, transforming them into indistinguishable encrypted forms while preserving data integrity for subsequent decryption. If adversarial perturbations remain visible in the encrypted output, attackers might identify these as vulnerabilities to exploit. The imperceptibility of encrypted adversarial images indicates that the encryption algorithm is robust, uniform, and resilient against data-dependent attacks. It shows that the algorithm not only secures the data but also disrupts the potential of adversarial patterns to undermine system integrity, providing an additional layer of security beyond traditional encryption methods.

### Chosen-plaintext attacks: all-black and all-white image analysis

Chosen-plaintext attacks (CPA) often exploit images with uniform pixel values, such as entirely black (0) or completely white (255), as these are among the most commonly used plaintexts for testing the predictability of encryption algorithms. These inputs give attackers a straightforward way to analyze the relationship between plaintext and ciphertext, aiming to infer patterns or weaknesses in the encryption process. Figure 25 illustrates the encryption and decryption results for all-black and all-white images. The encrypted version of the image exhibited no visible patterns or resemblance to the original. It ensures that attackers cannot derive useful information from the ciphertext to compromise the encryption keys, validating the algorithm’s robustness against predictability.

### MSE and PSNR

In image processing, the MSE is a statistic that is used to measure the difference between two images. It is calculated by equation (4) using double summation as follows:

4 $$\begin{aligned} \text {MSE} = \frac{1}{R \times C} \sum _{i=1}^{R} \sum _{j=1}^{C} (I_{\text {original}}(r, c) - I_{\text {distorted}}(r, c))^2 , \end{aligned}$$

where the image dimensions are indicated by $R$ and $C$, and $I_{\text {original}}(r, c)$ and $I_{\text {distorted}}(r, c)$ are the intensities of the pixel at row $r$ and column $c$ in the original and distorted (encrypted) images, respectively.

Another often-used metric to evaluate an image’s quality is the PSNR. It is calculated via equation (5) as follows:

5 $$\begin{aligned} \text {PSNR} = 10 \cdot \log _{10}\left( \frac{{\text {MAXIMUM}^2}}{{\text {MSE}}}\right) . \end{aligned}$$

The maximum possible pixel value in this case is represented by $\text {MAXIMUM}$ (e.g., 255 for an 8-bit image). The PSNR provides a logarithmic scale to show the relationship between the greatest potential power and the error power. It is measured in decibels (dB). The MSE and PSNR values for the RGB components of several images are displayed in Table 10.

**Table 10:** Image quality metrics (MSE and PSNR) for different encrypted images and their RGB components.

Testing encrypted image	MSE			PSNR (dB)		
	R	G	B	R	G	B
Multiple images	4348.2332	3377.4158	3268.5114	11.7477	12.8450	12.9873
Brain MRI	5707.8850	4455.6269	3683.5867	10.5611	11.6417	12.4681
Skin cancer	4735.3033	2857.8787	2555.8665	11.3773	13.5704	14.0554
Chest X-ray	4166.3107	3860.5932	3887.8420	11.9333	12.2643	12.2337
Baboon	2885.7602	2521.0127	3091.4646	13.5282	14.1151	13.2292

### NPCR and UACI

The amount of pixel modifications in an encrypted image that occur from a single pixel change in the original image is measured using a metric called the NPCR. Equation (6) formulates it as follows:

6 $$\begin{aligned} \text {NPCR} = \frac{1}{R \times C} \sum _{r=1}^{R} \sum _{c=1}^{C} \Delta (r, c) \times 100, \end{aligned}$$

where $\mathscr {C}_1, \text {and} \ \mathscr {C}_2$ are the encrypted images caused by a one-pixel change in the original image, and $\Delta (r, c)$ is a binary indicator, taking a value of 1 otherwise, and 0 when $\mathscr {C}_1(r,c) = \mathscr {C}_2(r,c)$. The multiplication by 100 highlights the rate per pixel change, and the summation is applied over the image’s dimensions.

The UACI offers insight into the average intensity of discrepancies between two encrypted images. It is calculated by equation (7) in the following manner:

7 $$\begin{aligned} \text {UACI} = \frac{1}{C \times R} \sum _{r=1}^{C} \sum _{c=1}^{R} \frac{|{\mathscr {C}_1(r,c) - \mathscr {C}_2(r,c)}|}{255} \times 100. \end{aligned}$$

In this context, $\mathscr {C}_1(r,c)$ and $\mathscr {C}_2(r,c)$ represent the pixel values in encrypted images before and after a single-pixel modification in the original image. The ideal values for NPCR and UACI for an ideally encrypted image are $99.61\%$ and $33.46\%$, respectively. Table 11 shows that the NPCR and UACI values for the RGB components of different images are around the theoretical value and thus secure against differential attacks. Additionally, the last two rows show values are passing the thresholds for encrypted images of adversarial images from FGSM and DeepFool, hence the adversarial attacks are unsuccessful.

**Table 11:** Image quality metrics (NPCR and UACI) for different encrypted images and their RGB components.

Testing encrypted image	NPCR %			UACI %		
	R	G	B	R	G	B
Multiple images	99.6025	99.5964	99.6384	33.4548	33.5090	33.4709
Brain MRI	99.6227	99.5770	99.6044	33.5441	33.4396	33.4387
Skin cancer	99.5880	99.6059	99.6075	33.4218	33.4246	33.5662
Chest X-ray	99.6113	99.5972	99.5869	33.4716	33.3856	33.3821
Baboon	99.5953	99.6056	99.6109	33.3729	33.5050	33.4605
Lena	99.7643	99.6365	99.7959	33.3643	33.4455	33.4514
Pepper	99.8612	99.6971	99.9468	33.4971	33.4638	33.4560
Breast Carcinoma	99.8390	99.7631	99.6113	33.4790	33.4971	33.4550
Gliomia	99.0463	99.4289	99.3301	33.5192	33.4928	33.5194
Barbara	99.7219	99.8459	99.7749	33.3863	33.4585	33.3626
Average of 10 images	99.6253	99.6354	99.6507	33.4511	33.4622	33.4563
Average of 50 random images	99.6228	99.6241	99.6231	33.4680	33.4535	33.4995
Adversarial attack (FGSM)	99.6215	99.6521	99.6338	33.5962	33.5786	33.5520
Adversarial attack (DeepFool)	99.6216	99.5850	99.5849	33.5435	33.4294	33.4823

### Statistical test for the NPCR

Following the methodology outlined in^72^, we evaluate the effectiveness of the proposed algorithm using statistical tests for the number of pixels change rate (NPCR). Given two encrypted images $\mathscr {C}_1$ and $\mathscr {C}_2$, each of size $512 \times 512$, we define the hypotheses ($H_0$ and $H_1$) at a significance level $\alpha$ for $N(\mathscr {C}_1, \mathscr {C}_2)$ in equation (8) as follows:

8 $$\begin{aligned} \left. \begin{array}{l} H_0: N(\mathscr {C}_1, \mathscr {C}_2) = \mu _N, \\ \\ H_1: N(\mathscr {C}_1, \mathscr {C}_2)< \mu _N. \\ \\ \text {We reject } H_0 \text { if } N(\mathscr {C}_1, \mathscr {C}_2) < N_{\alpha }^{*}; \text { otherwise, we accept } H_0. \\ \text { Here,} \ N_{\alpha }^{*} = \mu _N - \phi ^{-1}(\alpha ) \sigma _N = \frac{\left( F - \phi ^{-1}(\alpha )\sqrt{\frac{F}{RC}}\right) }{F+1}, \\ \mu _N = \frac{F}{F+1}, \\ \sigma ^2_N = \frac{F}{(F+1)^2 RC}, \\ \end{array} \right\} \end{aligned}$$

where *F* denotes the highest pixel value in the original image.

**Table 12:** Statistical test for the NPCR.

Testing encrypted-image	F = 255				
	$\mu _{N}$	$\sigma _{N}$	$N_{0.05}^{*}$	$N_{0.01}^{*}$	$N_{0.001}^{*}$
Numerical values	99.6094	0.0122	99.5893	99.5810	99.5717
Multiple images (99.6124)			$\checkmark$	$\checkmark$	$\checkmark$
Brain MRI (99.6013)			$\checkmark$	$\checkmark$	$\checkmark$
Skin cancer (99.6004)			$\checkmark$	$\checkmark$	$\checkmark$
Chest X-ray (99.5984)			$\checkmark$	$\checkmark$	$\checkmark$
Baboon (99.6039)			$\checkmark$	$\checkmark$	$\checkmark$

As observed from Table 12, the $N(\mathscr {C}_1, \mathscr {C}_2)$ values for all medical images, including multiple images, brain MRI, skin cancer, chest X-ray, and baboon, exceed the $N_{\alpha }^{*}$ values for $\alpha = 0.05$, 0.01, and 0.001. Thus, we accept the null hypothesis ($H_0$). Consequently, the NPCR values indicate that the proposed algorithm is robust and reliable.

### Statistical test for UACI

Similarly, based on^72^, we can demonstrate that the proposed algorithm is effective via statistical tests for the unified average changing intensity (UACI). Assuming that we have two encrypted images $\mathscr {C}_1$ and $\mathscr {C}_2$, each of size $512 \times 512$, we define the hypotheses ($H_0$ and $H_1$) at a significance level $\alpha$ for $U(\mathscr {C}_1, \mathscr {C}_2)$ in equation (9) are as follows:

9 $$\begin{aligned} \left. \begin{array}{l} H_0: U(\mathscr {C}_1, \mathscr {C}_2) = \mu _U, \\ H_1: U(\mathscr {C}_1, \mathscr {C}_2) < \mu _U, \\ \text {We reject } H_0 \text { if } U(\mathscr {C}_1, \mathscr {C}_2) \notin (U_{\alpha }^{*+}, U_{\alpha }^{*-}); \text { otherwise, we accept } H_0. \\ \text { Here,} \ U_{\alpha }^{*+} = \mu _U + \phi ^{-1}(\alpha /2) \sigma _U, \\ U_{\alpha }^{*-} = \mu _U - \phi ^{-1}(\alpha /2) \sigma _U, \\ \mu _U = \frac{F+2}{3F+3}, \\ \sigma ^2_U = \frac{(F+2)(F^2 + 2F + 3)}{18(F+1)^2 RC}, \\ \end{array} \right\} \end{aligned}$$

where *F* is the highest pixel value in the original image.

**Table 13:** Statistical test for UACI.

Testing encrypted-image	F = 255				
	$\mu _{U}$	$\sigma _{U}$	$U_{0.05}^{*+}/U_{0.05}^{*-}$	$U_{0.01}^{*+}/U_{0.01}^{*-}$	$U_{0.001}^{*+}/U_{0.001}^{*-}$
Numerical values	33.4635	0.0462	33.3730 33.5541	33.3445 33.5826	33.3115 33.6156
Multiple images (33.4782)			$\checkmark$	$\checkmark$	$\checkmark$
Brain MRI (33.4741)			$\checkmark$	$\checkmark$	$\checkmark$
Skin cancer (33.4708)			$\checkmark$	$\checkmark$	$\checkmark$
Chest X-ray (33.4131)			$\checkmark$	$\checkmark$	$\checkmark$
Baboon (33.4461)			$\checkmark$	$\checkmark$	$\checkmark$

From Table 13, we observe that the $U(\mathscr {C}_1, \ \text {and } \ \mathscr {C}_2)$ values for all medical images, including multiple images, brain MRI, skin cancer image, chest X-ray image, and baboon image, fall within the interval $(U_{\alpha }^{*+}, U_{\alpha }^{*-})$ for $\alpha = 0.05$, 0.01, and 0.001. Thus, we accept the null hypothesis ($H_0$). Therefore, the UACI values indicate that the proposed algorithm is effective and reliable.

### NIST SP 800-22 Statistical test suite results

Here, we generated 18,000,000 bits and divided them into ten parts, each consisting of 1,800,000 bits. These batches were then analyzed via the NIST SP 800-22 statistical test suite. The results are presented in Table 14 below:

**Table 14:** The NIST SP 800-22 statistical test suite results on generated outputs of encrypted images.

Statistical Tests	P-Values	Result
Frequency	0.739918	$\checkmark$
Block Frequency	0.122325	$\checkmark$
Cumulative Sums : Forward	0.350485	$\checkmark$
Cumulative Sums : Reverse	0.213309	$\checkmark$
Non Overlapping Template Matching	0.350485	$\checkmark$
Overlapping Template Matching	0.122325	$\checkmark$
Longest Run	0.122325	$\checkmark$
Fast Fourier Transform	0.739918	$\checkmark$
Matrix Rank	0.911413	$\checkmark$
Serial 1	0.350485	$\checkmark$
Serial 2	0.739918	$\checkmark$
Universal	0.991468	$\checkmark$
Approximate Entropy	0.534146	$\checkmark$
Linear Complexity	0.534146	$\checkmark$

## Comparative analysis

In the realm of security analysis, it is imperative to compare methodologies and outcomes across various research papers to discern overarching trends, identify best practices, and pinpoint gaps in current knowledge. A robust comparative analysis typically focuses on contrasting the effectiveness, efficiency, and scalability of different security solutions or frameworks. Table 15 shows some comparisons with other medical image encryption papers.

**Table 15:** Comparison with other existing encryption algorithms.

Medical Image	Algo	Channel	NPCR	UPCR	IE	Correlation			
						Horizontal	Vertical	Diagonal	Average correlation
Baboon	^73^	R	99.5900	33.4600	7.9998	−0.0022	0.0022	−0.0019	−0.00059
		G	99.6000	33.4400	7.9998	−0.00017	−0.0004	−0.0005	−0.0012
		B	99.6100	33.4700	7.9998	−0.0006	−0.0054	−0.0032	−0.0012
	^74^	R	99.6536	33.4753	7.9972	-	-	-	−0.0008
		G	99.6078	33.5090	7.9972	-	-	-	0.0002
		B	99.6520	33.4176	7.9972	-	-	-	−0.0006
	^75^	R	99.6140	33.4843	7.9970	-	-	-	0.0013
		G	99.6073	33.4690	7.9978	-	-	-	0.0025
		B	99.6292	33.4965	7.9987	-	-	-	0.0010
	^76^	R	99.6118	33.4692	7.9898	-	-	-	-
		G	99.6084	33.4806	7.9897	-	-	-	-
		B	99.6156	33.5040	7.9895	-	-	-	-
	**Proposed**	R	99.5953	33.3729	7.9993	0.0128	0.0060	0.0036	−0.01994
		G	99.6056	33.5050	7.9993	0.0057	0.0171	−0.0166	0.0050
		B	99.6109	33.4605	7.9993	−0.0783	−0.0079	0.1031	0.035
Brain MRI	^77^	R	99.62959	47.97603	7.99928	0.00146	−0.00118	−0.00015	-
		G	99.62539	46.42358	7.99922	0.00072	0.00145	−0.00101	-
		B	99.61779	46.68882	7.99937	0.00242	0.00085	−0.00552	-
	^78^	R	99.5971	33.4136	7.99741	−0.00253	−0.00270	0.00211	-
		G	99.5819	33.4723	7.99756	−0.00372	−0.00147	−0.00031	-
		B	99.6032	33.4574	7.99711	−0.00834	0.01012	−0.00046	-
	**Proposed**	R	99.6227	33.5441	7.9993	0.0045	0.0197	0.0918	-
		G	99.5770	33.4396	7.9993	−0.0314	−0.0154	0.0185	-
		B	99.6044	33.4387	7.9992	−0.0114	−0.0014	−0.0231	-

## Conclusion

This paper presents a novel encryption technique that integrates DCGAN associated with the VPD approach to enhance the protection of medical images. The method uses a DL framework to generate a decoy image, which forms the basis for generating encryption keys using a timestamp, nonce, and 1-DEC map. The experimental results validate the efficacy of the approach in protecting medical images from various security threats, including unauthorized access, tampering, and adversarial attacks. Its computational complexity, $O(M \times N)$, ensures scalability for encrypting images of varying sizes, striking an effective balance between security and time efficiency as can be seen in tables 5 and 6. The large keyspace of $2^{440}$, derived from chaotic sequences with a precision of $10^{-16}$ and VPD XORing table, provides strong resistance against brute-force attacks and is comparable to other methods as shown in 7. The randomness of the keys and encrypted images are demonstrated through the NIST SP 800-22 statistical test suite provided in Tables 4 and 14, respectively. The robustness against key sensitivity, noise, and cropping attacks is shown in Figures 15-18, and 22-23. Security analysis results are shown (such as histogram plots in Figures 11-14 and correlation plots in Figures 19-21). The IE ($7.9993 \pm 0.0001$), correlation coefficient ($\pm 0.09$), MSE($4166.3107 \pm 1645.2980$), PSNR ($12.2643 \pm 1.7032$), NPCR ($99.60\% \pm 0.2\%$), and UACI ($33.47\% \pm 0.1\%$) underscore the high security and reliability of the encrypted images, as shown in Tables 8-11. Furthermore, the statistical NPCR and UACI are calculated in Tables 12 and 13, respectively. The proposed algorithm is also compared with existing algorithms, and compared values are provided in Table 15. The data presented in Tables 4-15 highlight the practical viability and superior security of the proposed approach.

In the future, expanding the framework to video encryption presents another promising avenue. Recent advances in Selective Video Encryption Algorithms (SVEA)^30^ and temporal action segmentation^79^ highlight methods for optimizing security and efficiency by focusing on critical video frames or segments. These approaches, which use techniques like chaotic maps for one-step encryption, reduce computational overhead while maintaining high security, making them ideal for real-time applications in surveillance and streaming services. Moreover, selective video encryption principles could be adapted to optimize performance by prioritizing regions of interest within a video frame. Hybrid chaotic systems and advanced neural network architectures could be employed to provide security across diverse multimedia datasets^80^. These advancements, coupled with scalable algorithms like CP or Tucker decompositions^81^, MACH^82^, etc., for real-time tensor decompositions, provide a pathway to enhance the adaptability, security, and computational efficiency of the encryption framework against sophisticated attacks. Several GAN architectures, including Conditional GANs (CGANs) and Wasserstein GANs (WGANs), or Hybrid-GANs can also be adapted for specific data types, including images, music, and tabular data^83^.

## Data Availability

The datasets (i.e., cancer images) used for key generation in this work are publicly available on Kaggle (under the terms specified by the Kaggle platform). It can be accessed via the link provided in "Key generation procedure" .
